# The Role of Operating Conditions in the Precipitation of Magnesium Hydroxide Hexagonal Platelets Using NaOH Solutions

**DOI:** 10.1021/acs.cgd.3c00462

**Published:** 2023-08-08

**Authors:** Salvatore Romano, Silvio Trespi, Ramona Achermann, Giuseppe Battaglia, Antonello Raponi, Daniele Marchisio, Marco Mazzotti, Giorgio Micale, Andrea Cipollina

**Affiliations:** † Dipartimento di Ingegneria, Università degli studi di Palermo, Viale delle Scienze, 90128 Palermo, Italy; ‡ Institute of Energy and Process Engineering, ETH Zurich, 8092 Zurich, Switzerland; § Department of Applied Science and Technology, Institute of Chemical Engineering, Politecnico di Torino, 10129 Torino, Italy

## Abstract

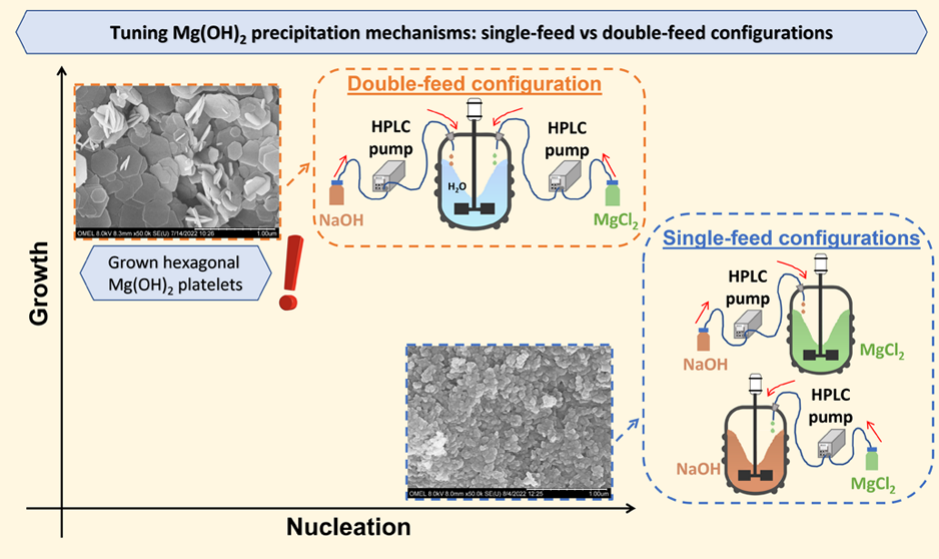

Magnesium hydroxide, Mg(OH)_2_, is an inorganic compound extensively employed in several industrial sectors. Nowadays, it is mostly produced from magnesium-rich minerals. Nevertheless, magnesium-rich solutions, such as natural and industrial brines, could prove to be a great treasure. In this work, synthetic magnesium chloride and sodium hydroxide (NaOH) solutions were used to recover Mg(OH)_2_ by reactive crystallization. A detailed experimental campaign was conducted aiming at producing grown Mg(OH)_2_ hexagonal platelets. Experiments were carried out in a stirred tank crystallizer operated in single- and double-feed configurations. In the single-feed configuration, globular and nanoflakes primary particles were obtained, as always reported in the literature when NaOH is used as a precipitant. However, these products are not complying with flame-retardant applications that require large hexagonal Mg(OH)_2_ platelets. This work suggests an effective precipitation strategy to favor crystal growth while, at the same time, limiting the nucleation mechanism. The double-feed configuration allowed the synthesis of grown Mg(OH)_2_ hexagonal platelets. The influence of reactant flow rates, reactant concentrations, and reaction temperature was analyzed. Scanning electron microscopy (SEM) pictures were also taken to investigate the morphology of Mg(OH)_2_ crystals. The proposed precipitation strategy paves the road to satisfy flame-retardant market requirements.

## Abstract 2

Hexagonal magnesium hydroxide crystals were successfully synthesized using magnesium chloride and sodium hydroxide solutions without any post-treatment. This was achieved thanks to the accurate control of the supersaturation level in a double-feed semi-batch reactor. The approach can be exploited to manage nucleation and crystal growth mechanisms in the extremely fast precipitation processes of metal hydroxides.

## 1 Introduction

In 2011, the European Union (EU) published the first list of high-supply risk “Critical Raw Materials”, CRMs,^1^ for the economy and social development of the EU. Magnesium, included among CRMs, is a shiny gray metal. EU imports more than 93% of magnesium from China. To tackle the EU mineral resource scarcity, alternative sources must be identified. In this context, the sea salt manufacturing process in saltworks generates a byproduct waste stream, called brine or bittern. Saltworks bitterns are characterized by a very high magnesium concentration reaching values up to 60 g/L, about 40 times higher than that in seawater (1.1–1.7 g/L).^2^ The high magnesium content makes bitterns excellent candidates for the recovery of this element, turning waste into a treasure.

A winning strategy is to extract magnesium in the form of magnesium hydroxide, Mg(OH)_2_, via reactive crystallization.^3,4^ Magnesium hydroxide is a white and odorless compound extensively employed as (i) an excipient for pharmaceutical and nutraceutical products; (ii) an acidic waste neutralizer thanks to its adsorptive and coagulative properties, (iii) a precursor for magnesium oxide and magnesium carbonate production, and (iv) a smoke-suppressing flame-retardant agent in composite polymeric materials.^5,6^ Depending on the industrial application, Mg(OH)_2_ products must fulfill specific requirements. As an example, flame-retardant applications require Mg(OH)_2_ hexagonal platelets characterized by an average particle size of ∼0.5 to 1.5 μm and a specific surface area of less than 10 m^2^/g.^7^ These characteristics have been typically achieved by adopting solvo/hydrothermal and microwave heating treatments.^8,9^ Hydrothermal synthesis, however, requires relatively long reaction times (from 6 to 24 h or more) and high temperatures (up to 200 °C).^10^ Several studies have dealt with the possibility to synthesize Mg(OH)_2_ hexagonal platelets via reactive crystallization using aqueous ammonia solution, NH_4_OH, or sodium hydroxide solutions.^11−14^ Mg(OH)_2_ hexagonal platelets have been synthesized using NH_4_OH, while, to the best of the authors’ knowledge, globular or flakes nano-primary particles have been reported employing only NaOH solutions.^15,16^ However, low Mg^2+^ conversions are typically attained when using NH_4_OH solutions. Further, the presence of ammonium ions (byproducts) makes the suspensions dangerous especially if these are employed in electrolytic processes.^17^ Conversely, NaOH solutions guarantee a 100% Mg^2+^ conversion with no dangerous byproducts.^2^

When using NaOH solutions, the Mg(OH)_2_ precipitation process has been reported to be characterized by very fast nucleation kinetics, considerably higher than growth kinetics even at low reactant concentrations of about 2 mM.^18^

In the process, reactants’ mixing is the rate-determining step affecting the final produced particle features.^19,20^ Battaglia et al.^21^ investigated the influence of mixing on Mg(OH)_2_ particles precipitated from synthetic 1 M MgCl_2_ and 2 M NaOH solutions employing two T-mixers. An analytical method was followed to characterize Mg(OH)_2_ particle assembly state by treating Mg(OH)_2_ suspensions with ultrasound and adding the poly(acrylic acid, sodium salt) as a dispersant. Tai et al.^22^ synthesized nanosized round and disk-shaped Mg(OH)_2_ particles in a spinning disk reactor using 0.2–0.92 M MgCl_2_ and 0.4–1.84 M NaOH solutions. The authors analyzed the influence of rotation speed, reactant flow rates, and concentrations on the produced Mg(OH)_2_ particles. Synthesized Mg(OH)_2_ powders were dispersed in water with the aid of a sonicator also adding poly(acrylic acid, sodium salt) and sodium hexametaphosphate as dispersants. Shen et al.^23^ explored the Mg(OH)_2_ precipitation in a novel impinging stream-rotating packed bed reactor employing 0.25–1.25 M MgCl_2_ and 0.5–2.5 M NaOH solutions. Particles were analyzed after dispersion in distilled water by sonication adding only a sodium hexametaphosphate solution as a dispersant. Ren et al.^7^ synthesized Mg(OH)_2_ particles from 1 M MgCl_2_ and 2 M NaOH solutions in a T-type microchannel reactor at 70 °C.

Mg(OH)_2_ precipitation was also investigated in stirred tank reactors. Mullin et al.^24^ precipitated Mg(OH)_2_ by mixing MgCl_2_ and NaOH solutions in a single-feed batch reactor. The authors studied the influence of different reactant concentrations (Mg^2+^ = 12.5, 25, and 50 mM) and stirring speeds (250, 500, and 700 rpm). Wu et al.^25^ performed Mg(OH)_2_ precipitation using MgCl_2_ and NaOH in a single-feed semi-batch reactor. The authors also treated the samples by hydrothermal treatment in the presence of 1 g/L of calcium chloride to increase the particle size and improve their morphology.

Overall, in all of the above-discussed studies, with the exception of the hydrothermal case, only Mg(OH)_2_ globular nanometric primary particles (50–200 nm) were always identified.

To favor crystal growth in the precipitation process of sparingly soluble compounds, Stavek et al.^26^ conducted the precipitation of silver halide compounds in a stirred beaker, feeding the reagents using controlled double jets. Song et al.^16^ tried to synthesize hexagonal magnesium hydroxide crystals in a single- and double-feed batch reactor under vigorous stirring. Highly concentrated MgCl_2_ solutions (2–4.5 M) and stoichiometric NaOH solution were added to a NaCl solution (2–3.5 M). Micro-sized ball-like aggregate/agglomerates particles were identified. Henrist et al.^11^ also performed Mg(OH)_2_ precipitation tests in a double-feed mode system exploring the influence of (i) alkaline solution types, i.e., NaOH or NH_4_OH; (ii) types of counterions, i.e., Cl^–^, NO_3_^–^ or SO_4_^2–^; and (iii) reaction temperature. Globular ∼300 nm cauliflower particles were precipitated from NaOH solutions at 60 °C, while ∼360 nm platelet-shaped crystals were obtained with NH_4_OH. No Mg(OH)_2_ hexagonal platelets were achieved in both studies.

This short literature review clearly highlights that despite several attempts, no hexagonal platelet Mg(OH)_2_ crystals precipitated from MgCl_2_ solutions using NaOH have ever been reported.

The present work aims at filling this gap by thoroughly analyzing the magnesium hydroxide precipitation process in single- and double-feed semi-batch crystallizers. Several operating conditions were explored, aiming at triggering crystal growth using NaOH solutions. These Mg(OH)_2_ particles would be of high interest in view of recovering Mg(OH)_2_ from natural wastes as saltworks bitterns. In the experimental campaign, synthetic MgCl_2_ and NaOH solutions were employed and the effect of reactant flow rates, reactant concentrations, and reaction temperature was investigated. Synthesized Mg(OH)_2_ products were characterized in terms of size, morphology, and surface area properties. For the first time in the literature, Mg(OH)_2_ platelet crystals were obtained using relatively concentrated MgCl_2_ and NaOH solutions without any dispersant addition or modification treatment. This result was achieved by accurately controlling the operating conditions in a double-feed semi-batch stirred reactor. The relevance of the here presented experimental campaign does not only apply to the case of the recovery of Mg(OH)_2_ from brines. The accurate control of the supersaturation level in a double-feed semi-batch stirred reactor, marked in the present work, can be extended to the precipitation of sparingly soluble compounds, whose precipitation processes are characterized by very fast kinetics.

## 2 Materials and Methods

### 2.1 Experimental Setup

**Figure 1:**
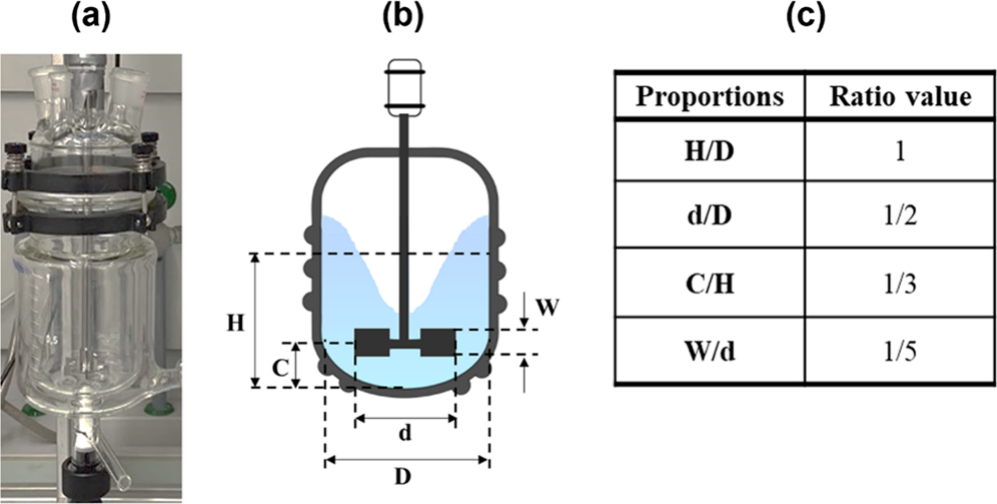
(a) Picture of the employed crystallizer; (b) schematic representation of the employed Mg(OH)_2_ unbaffled stirred tank crystallizer, and (c) geometrical proportions reflecting the standard geometry of the stirred tank.

Mg(OH)_2_ precipitation tests were carried out in a jacketed glass unbaffled stirred tank reactor, depicted in Figure 1a. An unbaffled configuration was chosen to prevent any undesirable secondary phenomena, e.g., secondary nucleation events at the baffles.^27,28^ Theoretical and empirical correlations have been proposed in the literature to estimate mixing times in unbaffled stirred tanks, adopting specific geometrical reactor configurations, referred to as the standard geometry.^29,30^ In the present experimental campaign, the adopted jacketed reactor, whose schematic representation and geometry proportions are reported in Figure 1b,c, had a 0.100 m diameter, *D*, with a round bottom. A six-blade Rushton turbine of 0.050 m diameter, *d*, and 0.010 m blade height, *W*, was employed and placed at 0.033 m, *C*, from the bottom of the tank. In all experiments, the liquid height, *H*, at the end of the test was 0.100 m, equivalent to a volume of 0.785 L. All geometry features were chosen to be complying with a typical standard geometry investigated in the literature.^29^

To comply with the standard geometry proportions, experiments were started with an initial volume of 0.685 L, equivalent to a liquid height of 0.087 m. After adding a volume of reactants of 0.100 L, the total volume increased by less than 15%, reaching the standard 0.100 m level. This strategy allowed the ratio *H*/*D* to be roughly maintained during all experimental tests. The stirring speed was set equal to 400 rpm to guarantee a good mixing yet avoiding any air bubbles entraining from the vortex. Under the adopted operating conditions, the tip speed and the specific power input were ∼1 m/s^31^ and 92 W/m^3^,^29^ respectively. The mixing time in the adopted stirred reactor was estimated to be about 15 s, following the correlation reported by Scargiali et al.^29^

**Figure 2:**
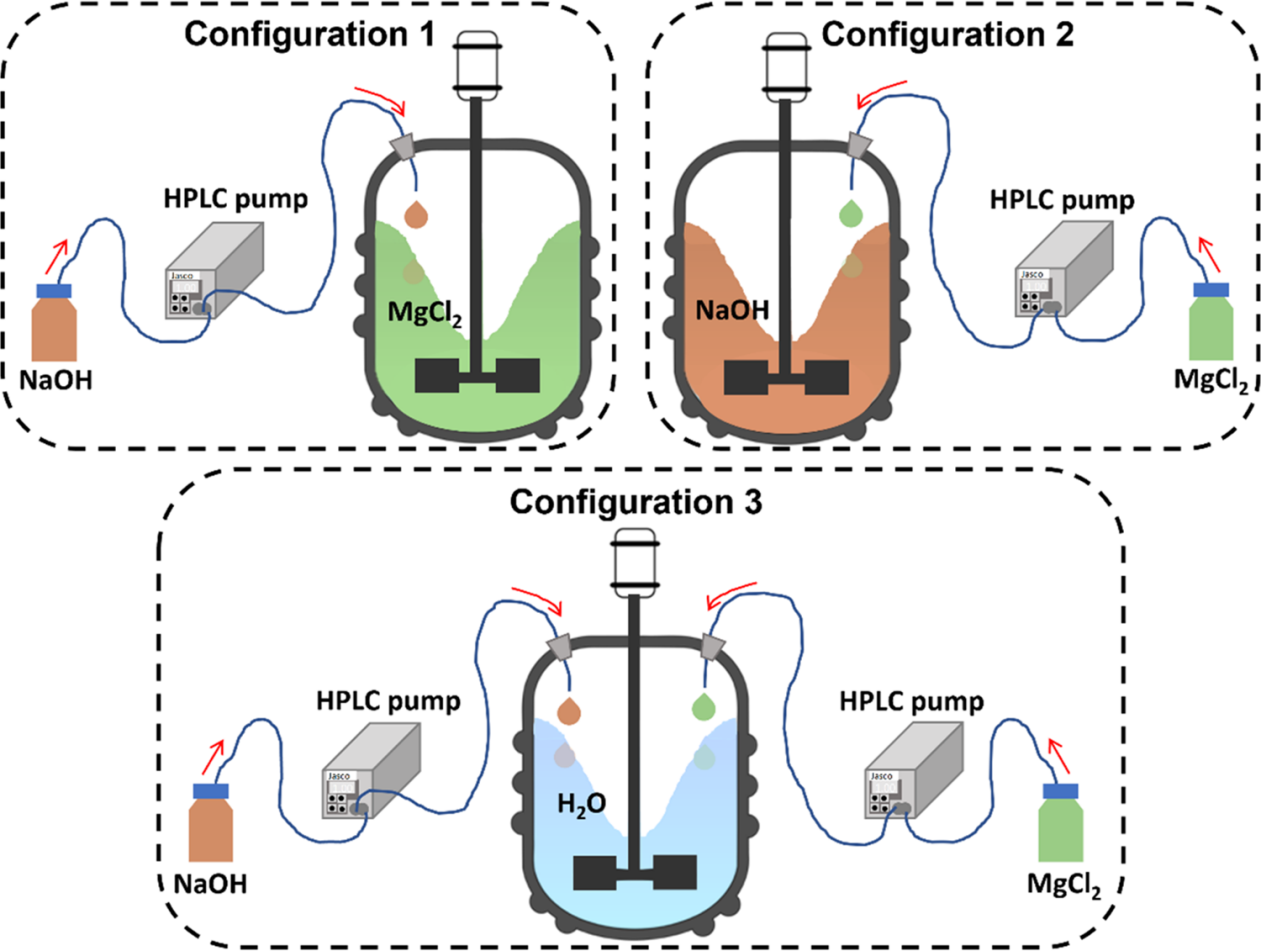
Schematic representation of the three employed configurations. High-performance liquid chromatography (HPLC) pumps were used to feed the solutions.

Mg(OH)_2_ precipitation tests were conducted following two different semi-batch operating strategies: (i) a single-feed and (ii) a double-feed. In the single-feed mode, a solution volume of 0.100 L of one of the reactants was fed into a 0.685 L solution of the other one; conversely, in the double-feed arrangement, 0.050 L of MgCl_2_ and NaOH solutions, for a total volume addition of 0.100 L, were pumped into 0.685 L ultrapure water bath. Figure 2 is a schematic representation of the two operating strategies resulting in 3 configurations: (i) Configurations 1 and 2, where either NaOH or MgCl_2_ solutions were pumped in single-feed arrangement into either MgCl_2_ or NaOH baths, respectively, and (ii) Configuration 3, where NaOH and MgCl_2_ solutions were added into the reactor already filled with ultrapure water.

In all tests, magnesium chloride (MgCl_2_; Sigma-Aldrich BioXtra, ≥99.0%) and sodium hydroxide (NaOH; Sigma-Aldrich puriss. p.a., ACS reagent, *K* ≤ 0.02%, ≥98.0%) pellets were dissolved in ultrapure water (Milli-Q) to prepare the feed solutions. The use of analytical-grade reagents ensured a high purity of the synthesized Mg(OH)_2_ products. Moreover, Mg(OH)_2_ always crystallizes in the trigonal *P*3̅*m*1 space group.

Magnesium and hydroxyl ions concentrations were checked by complex titration with (i) ethylenediaminetetraacetic acid (EDTA), for Mg^2+^, and (ii) acid–base titration, for OH^–^. Reactants solutions were fed drop-wise using high-performance liquid chromatography (HPLC) pumps (JASCO PU-986). Most of the experiments were carried out at room temperature. For tests at 6 and 60 °C, the temperature (T) of the reaction environment was kept constant using a Huber Ministat waterbath. This device was equipped with a pumping system to handle the cooling or heating fluid (distilled water) in the plain jacket. All experiments were conducted adopting stoichiometric reactants solutions amount. The solution/suspension pH was monitored offline or inline by a pH probe (Metrohom Profitrode electrode), and its final value was always in the range between 10.4 and 10.6, thus ensuring the complete reagents’ conversion.

#### 2.1.1 Test Conditions

Table 1 lists the precipitation tests conducted in single- and double-feed modes. Four tests were carried out in single-feed modes (Configurations 1 and 2) studying the influence of reactants feeding flow rates. Conversely, the effect of different parameters was analyzed in the double-feed mode (Configuration 3), namely: (i) feed solutions flow rates, (ii) feed solutions concentrations, and (iii) reaction temperature. In Table 1, cases are grouped based on the adopted Configuration, i.e., 1, 2, or 3. The reproducibility and repeatability of the experimental data are discussed in the Supporting Information.

**Table 1:** Operating Conditions Adopted during Single- and Double-Feed Experiments^a^

setup config.	case	MgCl_2_ concentration [M]	NaOH concentration [M]	flow rate (*Q*) [mL/min]	feeding time (*t*_f_) [min]	temperature [°C]
1	1	0.036 ± 0.002	0.500 ± 0.025	1.00 ± 0.05	100	25 ± 1
	1.f1	0.036 ± 0.002	0.500 ± 0.025	0.500 ± 0.025	200	25 ± 1
2	2	0.250 ± 0.013	0.072 ± 0.004	1.00 ± 0.05	100	25 ± 1
	2.f1	0.250 ± 0.013	0.072 ± 0.004	0.500 ± 0.025	200	25 ± 1
3	3	0.500 ± 0.025	1.00 ± 0.05	0.500 ± 0.025	100	25 ± 1
	3.f1	0.500 ± 0.025	1.00 ± 0.05	0.250 ± 0.013	200	25 ± 1
	3.f2	0.500 ± 0.025	1.00 ± 0.05	1.00 ± 0.05	50	25 ± 1
	3.f3	0.500 ± 0.025	1.00 ± 0.05	5.00 ± 0.25	10	25 ± 1
	3.f4	0.500 ± 0.025	1.00 ± 0.05	7.50 ± 0.40	6.5	25 ± 1
	3.c1	0.125 ± 0.006	0.250 ± 0.013	0.500 ± 0.025	100	25 ± 1
	3.c2	0.250 ± 0.013	0.500 ± 0.025	0.500 ± 0.025	100	25 ± 1
	3.c3	1.00 ± 0.05	2.00 ± 0.10	0.500 ± 0.025	100	25 ± 1
	3.t1	0.500 ± 0.025	1.00 ± 0.05	0.500 ± 0.025	100	6 ± 1
	3.t2	0.500 ± 0.025	1.00 ± 0.05	0.500 ± 0.025	100	60 ± 1

a Quantity uncertainties were estimated by combining the dispersion observed between different trials (reproducibility error) and instrument uncertainties.

A reference case was chosen for each configuration, and additional letters were used to indicate the parameter varied in the tests, i.e., (*c*) reagent concentration, (*t*) temperature, and (*f*) flow rate. After the letters, a number was added to distinguish cases at a fixed parameter. The range of parameters was chosen as follows: (i) reference cases, namely, Cases 1, 2, and 3, were characterized by the same final ∼1.4 g mass of precipitate; (ii) Mg^2+^ concentrations embraced values from that of seawater (∼0.06 M) to real brine (∼1.00 M); (iii) reactant flow rates were typical of lab scale studies; and (iv) the reaction temperature range was identified according to operational limits of the Huber Ministat waterbath device.

### 2.2 Mg(OH)_2_ Particle Characterization Strategy

#### 2.2.1 Particle Size Distributions: Static and Dynamic Light Scattering Techniques

Mg(OH)_2_ particle size distributions (PSDs) were measured by static (SLS) and dynamic light scattering (DLS) techniques. All measurements were performed with and without ultrasound treatment (US) and the addition of the dispersant poly(acrylic acid, sodium salt), (PAA, MW1200, Sigma-Aldrich, Inc.), to accurately characterize Mg(OH)_2_ particles reducing their agglomeration influence.^11,21^ At least 5 volume-PSD measurements for each sample were performed.

The SYMPATEC HELOS granulometer (R3 LENS, *f* = 100 mm, 0.5/0.9–175 μm) was employed to characterize particle sizes in the range of 0.7–175 μm, while the Zetasizer Nano ZS (Malvern Instruments, U.K.) was adopted for particle sizes measurements between 0.010 and 3 μm. The Mg(OH)_2_ refractive index was set at 1.58.

Specifically:

(1) Mg(OH)_2_ particles larger than 0.7 μm were characterized using the SYMPATEC HELOS granulometer. This device was equipped with a SUCELL wet dispersion system having a small volume adapter (∼50 mL) provided with a stirrer for sample homogenization. The pump speed was set at 50% following the SUCELL user guide. In the absence of ultrasound treatment and PAA addition, measurements were carried out as follows: (1) the volume adapter was filled with ultrapure water reaching a volume of ∼50 mL; (2) Mg(OH)_2_ slurry was gradually added until an obscuration between 15 and 20% was reached, corresponding to a solids concentration of about 0.3 g/L. Conversely, when adopting PAA and ultrasound, a different procedure was followed: (1) samples were diluted in ultrapure water until reaching a solid concentration of about 0.3 g/L; (2) the PAA dispersant was added until reaching a concentration of 4.9 g/kg in the diluted suspension; (3) samples were exposed to an ultrasonic bath (Elma Elmasonic S 40 H (220–240 V), ultrasonic frequency of 37 kHz) for 5 min; and (4) the diluted samples were loaded into the small volume adapter (∼50 mL) to perform the analyses.
(2) Mg(OH)_2_ particles smaller than 1 μm were characterized using the Malvern Zetasizer Nano ZS device. In the absence of ultrasound treatment and PAA addition, measurements were carried out as follows: (1) samples were diluted in ultrapure water until reaching a solid concentration of 0.3 g/L, as suggested by the user guide; (2) the diluted suspension was loaded into a disposable cuvette and the analysis was carried out. Conversely, when adopting PAA and ultrasounds, measurements were conducted as follows: (1) samples were diluted in ultrapure water until reaching a solid concentration of 0.3 g/L; (2) the PAA dispersant was added until reaching a concentration of 4.9 g/kg in the diluted suspension; (3) samples were exposed to an ultrasonic bath (Elma Elmasonic S 40 H 220–240 V, ultrasonic frequency of 37 kHz) for 5 min; and (4) the diluted suspension was loaded into a disposable cuvette and the analysis was carried out.

In all cases, volume PSDs measurements were performed within 10 min after completion of the precipitation tests. For all of the collected volume PSDs, their median diameters, namely, *d*(0.5), were determined. The *d*(0.5) is the median diameter that halves the volume distribution, i.e., 50% of the particles lie below and above the *d*(0.5) value. Average median diameters and their standard deviations (calculated among 5 measurements) are discussed in Section 3, while, for the sake of completeness, average volume PSDs are reported in Appendix A.

The SYMPATEC HELOS granulometer and the Zetasizer Nano ZS are based on static light scattering and dynamic light scattering techniques, respectively. Different PSDs for the same sample can be obtained by adopting the two techniques. In the Supporting Information, the possible results offset is discussed considering PSDs of Case 3 and Case 3.c1.

#### 2.2.2 Morphological Analyses: Scanning Electron Microscopy (SEM) Technique

Mg(OH)_2_ particle morphology was assessed by scanning electron microscopy (SEM) technique using a Hitachi S-4800 scanning electron microscope. Samples were prepared as follows: (1) Mg(OH)_2_ suspensions were filtered with a Whatman GF/A glass microfiber filters (pore size of 1.6 μm) using a Büchner system; (2) cakes were washed with ultrapure water (Milli-Q) to remove residual sodium chloride traces (reaction byproducts); (3) cakes were dried in an oven at 120 °C for 24 h; (4) dry cakes were finally crushed with mortar and pestle; and (5) a platinum–palladium alloy coating was applied to make samples conductive. In the Supporting Information, SEM micrographs at different locations of the same sample are provided to demonstrate the relevance of the observations made in Section 3.

#### 2.2.3 Specific Surface Area Analyses: Brunauer–Emmett–Teller (BET) Technique

The specific surface area of Mg(OH)_2_ particles was determined by Brunauer–Emmett–Teller (BET) analyses (TriStar II Plus, Micromeritics). Samples were prepared following the same procedure as that for SEM analysis (Section 2.2.2). Before BET measurements, solids were degassed in a nitrogen environment for 3 h at 180 °C.

## 3 Results and Discussion

In this section, the results of Mg(OH)_2_ precipitation tests are presented. A preliminary discussion is made regarding the reaction environment conditions attained in the three reactor configurations. To distinguish the nature and origin of different Mg(OH)_2_ particles, the following nomenclature has been adopted:

- primary particles are single crystals;
- aggregates consist of many primary particles, linked by strong chemical bonds that cannot be broken by neither fluid shear stresses nor sonication;
- agglomerates are made of primary particles, aggregates, or a mixture of the two, bonded together by electrostatic forces. Agglomerates are weaker than aggregates and can be unpacked by physical treatments such as sonication.^32^

### 3.1 Preliminary Estimation of Supersaturation in Different Reactor Configurations

The driving force of a crystallization process is supersaturation (*S*), which is related to the actual concentration of a solute in a solution with respect to its solubility value.

In the Mg(OH)_2_ precipitation process, Mg^2+^ ions react with hydroxyl ones (OH^–^) provided by an alkaline reactant, i.e., NaOH. The driving force of the process is the supersaturation that is established when the solute concentration exceeds its solubility value. In the present study, supersaturation (*S*) is expressed in relative form as the difference between the product of ions activity and the solubility product (*k*_sp_) divided by *k*_sp_, as follows^18^
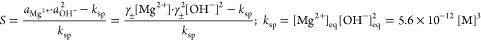
1where γ_±_ is the activity coefficient for multicomponent salt solutions that can be calculated by exploiting equations provided by Bromley.^33^

In the present experimental investigation, Mg^2+^ and OH^–^ ions were added in single- and double-feed modes, as schematically represented in Section 2, see Figure 2. In single-feed mode (Configurations 1 and 2, Figure 2), a drop of one reactant immediately gets in contact with the other reagent present in the liquid volume. Due to the very low Mg(OH)_2_ solubility, a significant local supersaturation level is reached, inducing the fast Mg(OH)_2_ precipitation before the complete dilution of the reagent droplet in the whole liquid volume. In this case, nucleation of nanometric particles, prone to aggregate rather than grow, occurs, as largely discussed in the literature.^34^

In the double-feed mode (Configuration 3, see Figure 2) conversely, the two reactants’ drops are fed into a water bath. If reactants are added from two opposite sites, as performed in the present work, reactants most likely dilute into the water bath before they meet and react. In this case, lower supersaturation levels can be foreseen with respect to the single-feed mode. On this basis, the possible order of magnitude of the initial supersaturation levels attained at the beginning of the experiments for the investigated configurations was estimated and is reported in Table 2. An instantaneous reaction between reactant drops and solution bath was assumed for the single-feed mode, while a dilution of the reactant drop (volume of drop equal to 0.050 mL) was considered to occur in a volume equivalent to 5, 10, and 20% of the reactor total volume (0.685 L).

**Table 2:** Estimation of Supersaturation Levels for the Three Investigated Configurations^a^

setup config.	1		2		3			
case	1		2		3			
	before dilution	after dilution	before dilution	after dilution	before dilution	after dilution (5%)	after dilution (10%)	after dilution (20%)
MgCl_2_ conc. [M]	0.036		0.250		0.500	7.30 × 10^–4^	3.65 × 10^–4^	1.82 × 10^–4^
NaOH conc. [M]	0.500		0.072		1	14.6 × 10^–4^	7.30 × 10^–4^	3.65 × 10^–4^
activity coeff. (γ_±_)	0.45		0.35		0.30	0.88	0.91	0.93
supersat. (*S*)	1.5 × 10^8^		1.0 × 10^7^		2.4 × 10^9^	1.9 × 10^2^	2.5 × 10^1^	2.5 × 10^0^

a A reactant drop of 50 μL was assumed to react as soon as it meets the other reagent in single-feed modes (Configurations 1 and 2), while in the double-feed mode, reactants drops were estimated to dilute in a volume equivalent to 5, 10, and 20% of the total volume (0.685 L) before reaction (Configuration 3).

In single-feed modes, the initial supersaturation values are expected to be very high (e.g., 10^7^–10^8^) due to the instantaneous contact between the two reagents see Table 2. Therefore, Mg(OH)_2_ precipitation will occur immediately at high reactant concentrations. Conversely, lower supersaturation values are expected in double-feed mode, favored by the high dilution factor in the water bath, leading the Mg(OH)_2_ precipitation to occur in milder conditions (namely, 1.9 × 10^2^, 2.5 × 10^1^, and 2.5 × 10^0^ considering a drop dilution in a volume equivalent to 5, 10, and 20% of the total reactor volume, respectively). In addition, Table 2 reports a case for the double-feed configuration, where no dilution was assumed. The calculated supersaturation level (2.4 × 10^9^) is the highest among the analyzed cases; however, it is most likely overestimated. As a matter of fact, the two reagent drops cannot react immediately since they dilute in the water bath as they are fed at two opposite reactor sides.

It must be stressed that supersaturation values reported in Table 2 were only estimated based on possible scenarios that could occur in the two investigated feeding mode systems. A more detailed description of the phenomena characterizing the analyzed systems can be provided by coupling computation fluid dynamic (CFD) simulations and population balance equations. Modeling tools can provide a deep interpretation of the influence of mixing conditions, reactant addition rates, reaction time, and reaction evolution on particle sizes, particle morphology, and level of agglomeration/aggregation during the experimental tests.

### 3.2 Mg(OH)_2_ Particles Characteristics

The influence of the feeding operating conditions on Mg(OH)_2_ particles characteristics (median diameters, *d*(0.5), and morphologies) is introduced in the following sections. Enlarged SEM images are also available in Appendix A.

#### 3.2.1 Single-Feed Configurations

**Figure 3:**
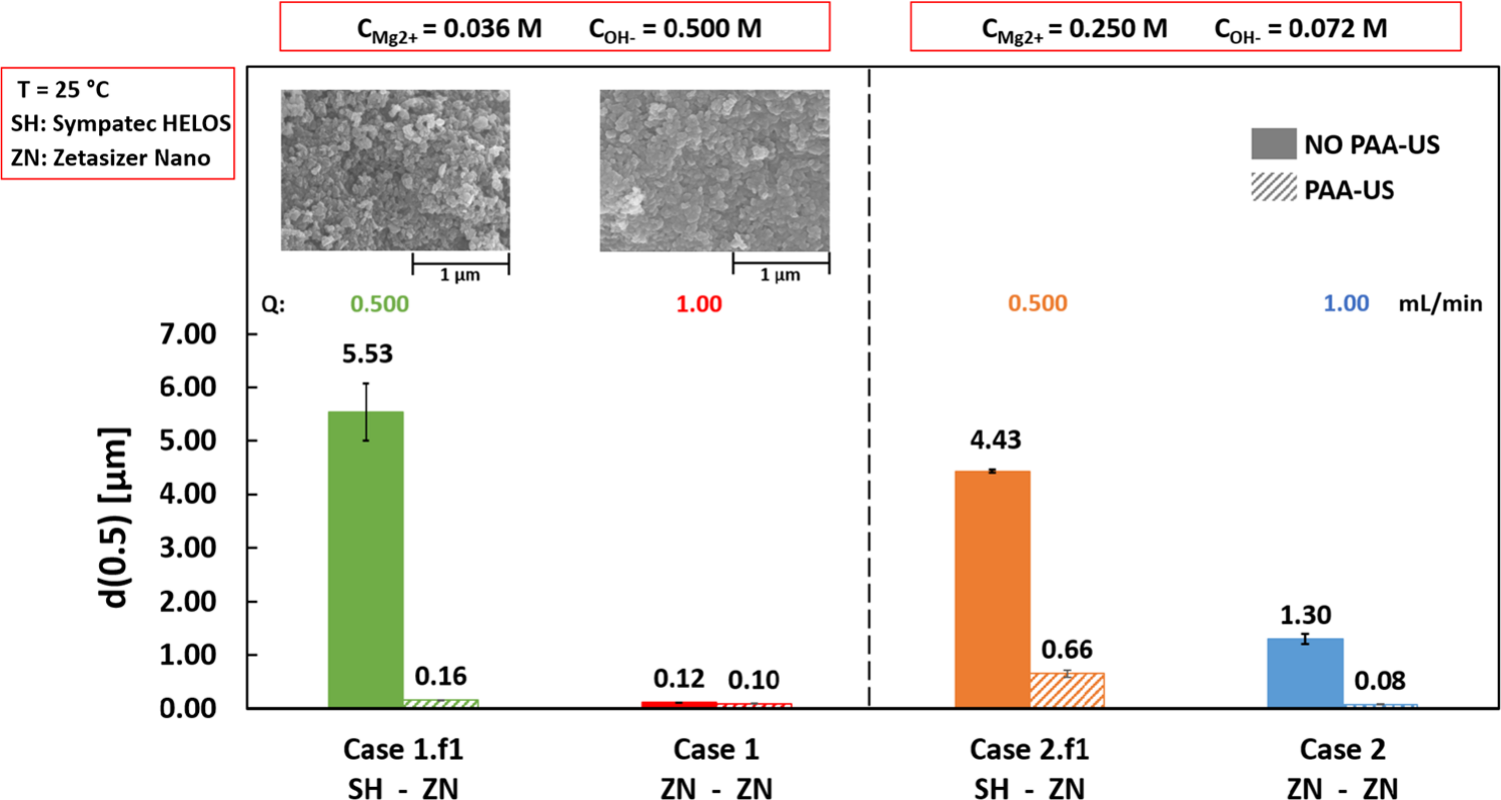
Median diameter *d*(0.5) values calculated before (solid bars) and after (diagonal stripes bars) PAA-US for single-feed cases. Configuration 1: 0.036 M MgCl_2_ and 0.500 M NaOH. Configuration 2: 0.250 M MgCl_2_ and 0.072 M NaOH. The bottom row indicates the employed particle size analyzer: Sympatec HELOS (SH) or Zetasizer Nano (ZN). The upper row reports SEM images.

Figure 3 shows the median diameter values obtained for Configurations 1 and 2 at reactant flow rates (*Q*) of 0.500 and 1.00 mL/min, see Table 1. For Configuration 1, SEM pictures were also reported. The employed MgCl_2_ and NaOH concentrations were (i) 0.036 and 0.500 M for Configuration 1 and (ii) 0.250 and 0.072 M for Configuration 2.

The reactant flow rate was 0.500 mL/min (Cases 1.f1 and 2.f1) and 1.00 mL/min (Cases 1 and 2). In Figure 3, solid and diagonal stripes bars refer to d(0.5) values calculated before and after PAA-ultrasound treatment (PAA-US), respectively.

At a feeding rate of 1 mL/min (solid red, Case 1, and blue, Case 2, bars), particles are more agglomerated in Case 2, as indicated by the *d*(0.5) shift before (solid bars) and after (diagonal stripes bars) PAA-US. Before sonication, *d*(0.5) is equal to ∼1.3 μm in Case 2, while it is ∼0.12 μm in Case 1. After sonication (diagonal stripes bars), the median diameters decrease down to ∼0.10 μm in both Cases. This suggests that the two different configurations produce similar Mg(OH)_2_ aggregates.

At a feeding rate of 0.500 mL/min, similar agglomerate sizes of ∼5.53 and 4.43 μm are observed for Case 1.f1 (solid green bar) and Case 2.f1 (solid orange bar), respectively. After sonication, aggregates are bigger in Case 2.f1 (∼0.66 μm, diagonal stripes orange bar) with respect to Case 1.f1 (∼0.16 μm, diagonal stripes green bar). Aggregates are always bigger than those produced at lower feeding time (higher flow rate). The bigger agglomerates observed in Case 2 with respect to Case 1 could be ascribed to the higher-pH environment attained during the reaction. At the beginning of the tests, the initial measured pH values were ∼10 and ∼13 for Case 1 and Case 2, respectively, due to the presence of MgCl_2_ or NaOH solutions in the reactor. In both cases, the final measured pH value was ∼10.5, close to the equilibrium one, indicating the complete conversion of the reagents. pH trends are reported in Appendix A. Particle stability in suspensions can be studied by ζ-potential analyses at different pH values. In the literature, Mg(OH)_2_ ζ-potential values have been reported to vary from +20 mV at pH 10 to −28 mV at pH 13.5, being null (isoelectric point) at pH ∼12.^35,36^ Therefore, the ζ-potential values are lower in Case 2 (pH from 13 to 10.5 passing through the isoelectric point) rather than in Case 1 (pH from 10 to 10.5), inducing a higher particle agglomeration. The pH influence is probably overcome by the higher residence time in Cases 1.f1 and 2.f1. The higher residence time may induce a higher particle collision probability leading to bigger agglomerates and aggregates.

For the sake of brevity, only SEM images of Case 1 and Case 1.f1 are reported in the upper row of Figure 3, since similar results were obtained in Cases 2 and 2.f1.

Nanometric globular/flakes primary particles can be observed in both cases. Unfortunately, it is quite difficult to distinguish if particles are more or less aggregated. The presence of globular/flakes particles was somehow expected. As discussed in Section 1, nanometric globular Mg(OH)_2_ particles are always produced by precipitation with NaOH solutions.^21−23^ This can be due to the high supersaturation levels reached during the tests, previously estimated for single-feed configurations. Consequently, nucleation and particle aggregation are the predominant phenomena over crystal growth.

#### 3.2.2 Double-Feed Configuration

The influence of (i) reagent flow rates, (ii) reagent concentrations, and (iii) reaction temperature was studied in the double-feed mode, as listed in Table 1.

Solid and diagonal stripes bars refer to *d*(0.5) values calculated before and after PAA-ultrasound treatment (PAA-US), respectively.

##### 3.2.2.1 Influence of Reactant Flow Rate in Double-Feed Configuration

**Figure 4:**
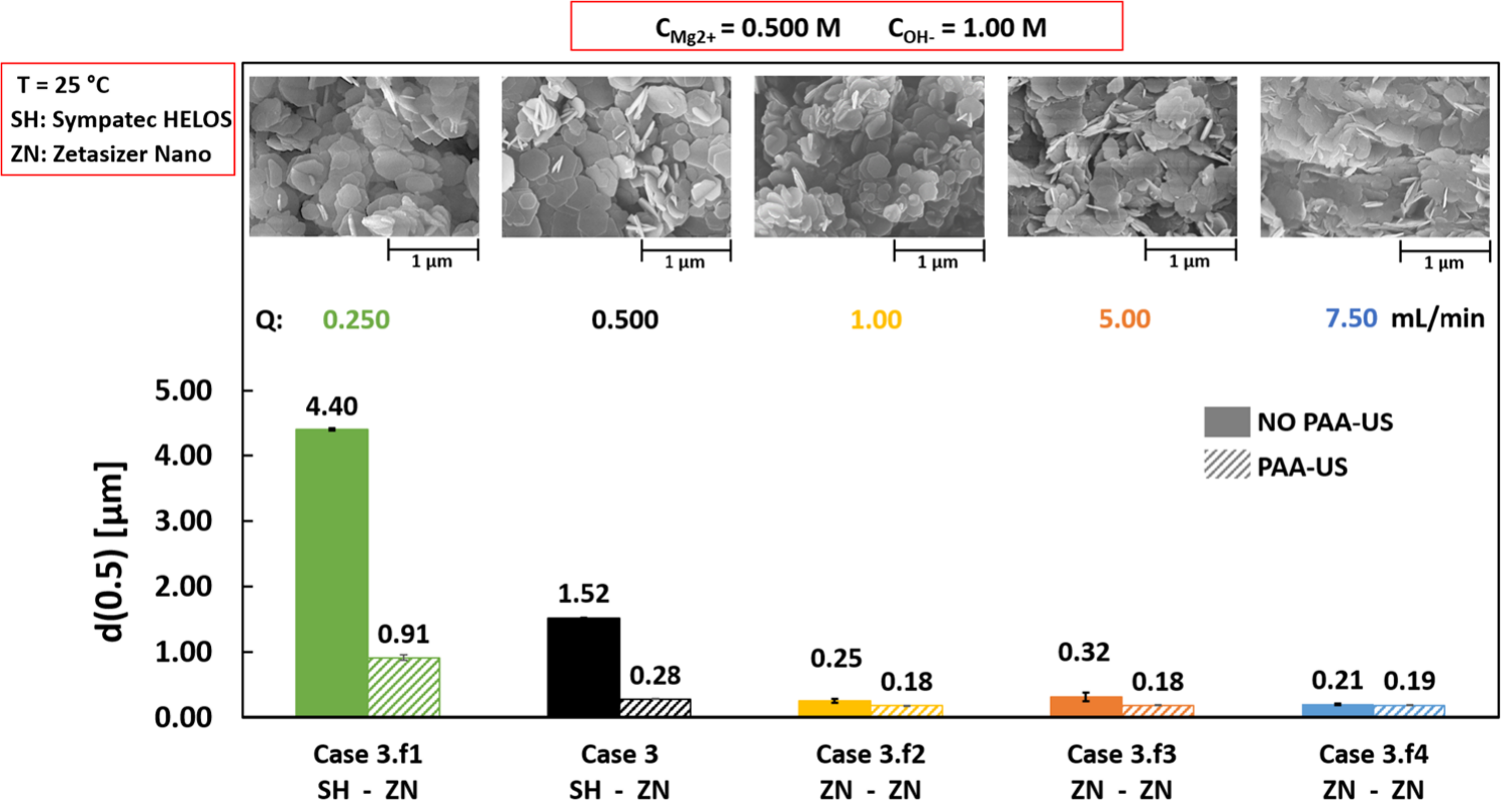
Effect of reagents feed flow rates on median diameter *d*(0.5) values calculated before (solid bars) and after (diagonal stripes bars) PAA-US. Flow rates of 0.250 mL/min (Case 3.f1), 0.500 mL/min (Case 3), 1 mL/min (Case 3.f2), 5 mL/min (Case 3.f3), and 7.50 mL/min (Case 3.f4). MgCl_2_ and NaOH concentrations = 0.500 and 1.00 M; stirring speed = 400 rpm; T = 25 °C. The bottom row indicates the employed particle size analyzer: Sympatec HELOS (SH) or Zetasizer Nano (ZN). The upper row reports SEM images.

Figure 4 reports median diameters for Cases 3.f1, 3, 3.f2, 3.f3, and 3.f4 obtained at feed flow rates of 0.250, 0.500, 1, 5.00, and 7.50 mL/min, respectively. In all tests, 0.500 M MgCl_2_ and 1.00 M NaOH solutions were used.

The upper row of Figure 4 shows SEM pictures for all cases.

Before PAA-US (solid bars), Cases 3.f2 (yellow), 3.f3 (orange), and 3.f4 (blue) are characterized by quite similar median diameters ranging between 0.21 and 0.32 μm. Conversely, bigger agglomerates, above 1 μm, are observed for Case 3 (solid black bar) and even bigger for Case 3.f1 (solid green bar). The same trend is also noticed after PAA-US (diagonal stripes bars), where bigger aggregates are observed for Cases 3.f1 (green) and 3 (black), clearly highlighting the influence of the feeding time on Mg(OH)_2_ particles. In particular, the higher the feeding time (moving from 6.5 min, Case 3.f4, to 200 min, Case 3.f1), the bigger are the particles. This behavior can be caused by stronger bridge formation between particles promoted by their higher residence time in the reactor. This behavior is in accordance with the results of Configurations 1 and 2. In all cases, particles are found to be characterized by a hexagonal platelets morphology, see the upper row in Figure 4. Mg(OH)_2_ platelets are better defined moving from the lowest to the highest reagent flow rates, i.e., from left to right in Figure 4.

This result can be ascribed to: (i) the higher reaction time that can promote crystal growth and (ii) the lower supersaturation level attained in the reactor favored by the slower addition of the reactants that can have more time to dilute before reacting. This is in accordance with the pH profile recorded for Case 3, see Appendix A.

In the literature, Mg(OH)_2_ hexagonal platelets have been reported only (i) using aqueous ammonia solution, (ii) adding additives, or (iii) after hydrothermal treatment due to the dissolution of small crystals allowing the growth of larger ones in the metastable zone (Ostwald ripening). The findings of Figure 4 indicate that the double-feed configuration allows accurate control of the supersaturation level in the precipitation process. The two reactants streams, fed into the water bath from two opposite sides, most likely dilute before they meet and react, thus guaranteeing low supersaturation levels. This is in accordance with the estimation of supersaturation levels presented in Section 3.1. As already mentioned in Section 1, the double-feed configuration can be of considerable interest for the controlled precipitation process of sparingly soluble compounds, such as halides, oxalates, and sulfates.^26,37,38^

##### 3.2.2.2 Influence of Reactant Concentrations in Double-Feed Configuration

**Figure 5:**
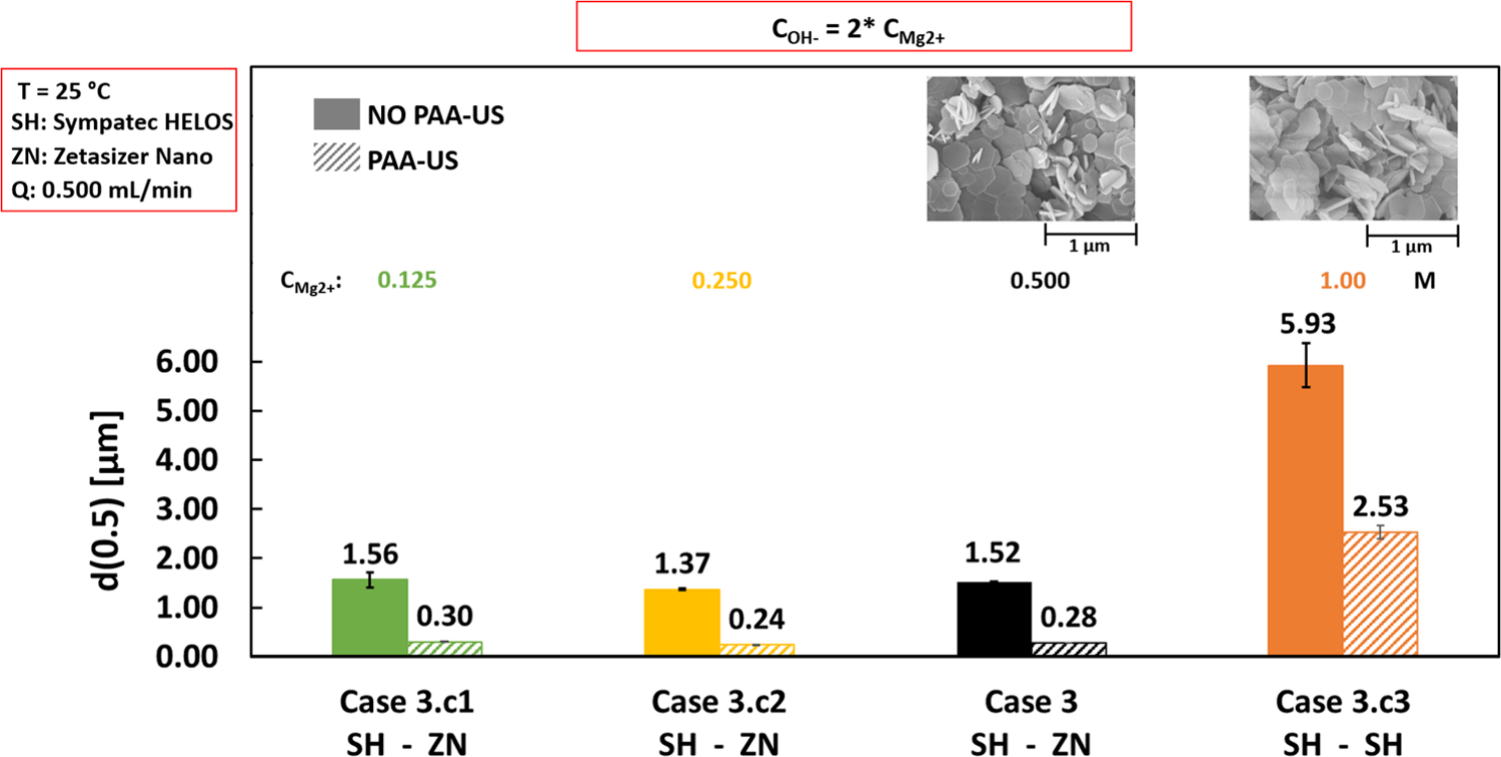
Effect of reactant concentrations on median diameter *d*(0.5) values calculated before (solid bars) and after (diagonal stripes bars) PAA-US. 0.125, 0.250, 0.500, and 1 M MgCl_2_ and their NaOH stoichiometric concentrations were employed for Cases 3.c1, 3.c2, 3, and 3.c3, respectively. Flow rates = 0.500 mL/min, stirring speed = 400 rpm, T = 25 °C. The bottom row indicates the employed particle size analyzer: Sympatec HELOS (SH) or Zetasizer Nano (ZN). The upper row reports SEM images.

Figure 5 shows the median diameters for double-feed Cases 3.c1, Case 3.c2, Case 3, and Case 3.c3 performed adopting MgCl_2_ concentrations of 0.125, 0.250, 0.500, and 1.00 M, respectively. Stoichiometric NaOH solution concentrations were always employed. The same feed flow rate of 0.500 mL/min and feeding time of 100 min were set in the experiments. For Cases 3 and 3.c3, SEM pictures are shown in the upper row of Figure 5.

Notably, the median diameters of Case 3.c3 (1.00 M MgCl_2_ and 2.00 M NaOH) show the biggest agglomerates and aggregates (solid and diagonal stripes orange bars in Figure 5) among the other cases. Cases 3.c1, 3.c2, and 3 exhibit quite similar median diameters: agglomerates of ∼1.37 to 1.52 μm (solid bars) and aggregates of ∼0.24 to 0.30 μm (diagonal stripes bars).

This can be explained by the Derjaguin–Landau–Verwey–Overbeek (DLVO) theory: particles have a higher tendency to agglomerate and aggregate in high-ionic-strength NaCl solutions (here the reaction byproduct) due to the compression of their electrical double layer.^39^

Interestingly, well-defined hexagonal platelets with a primary particle size of around ∼300 nm can be clearly observed in Case 3 and even in Case 3.c3, see Figure 5. The double-feed configuration guarantees very low supersaturation values also adopting the highest reactant concentrations (Case 3.c3). Therefore, no SEM images were collected for Cases 3.c1 and 3.c2, as they are expected to be similar to Case 3.

##### 3.2.2.3 Influence of Temperature on Double-Feed Configuration

**Figure 6:**
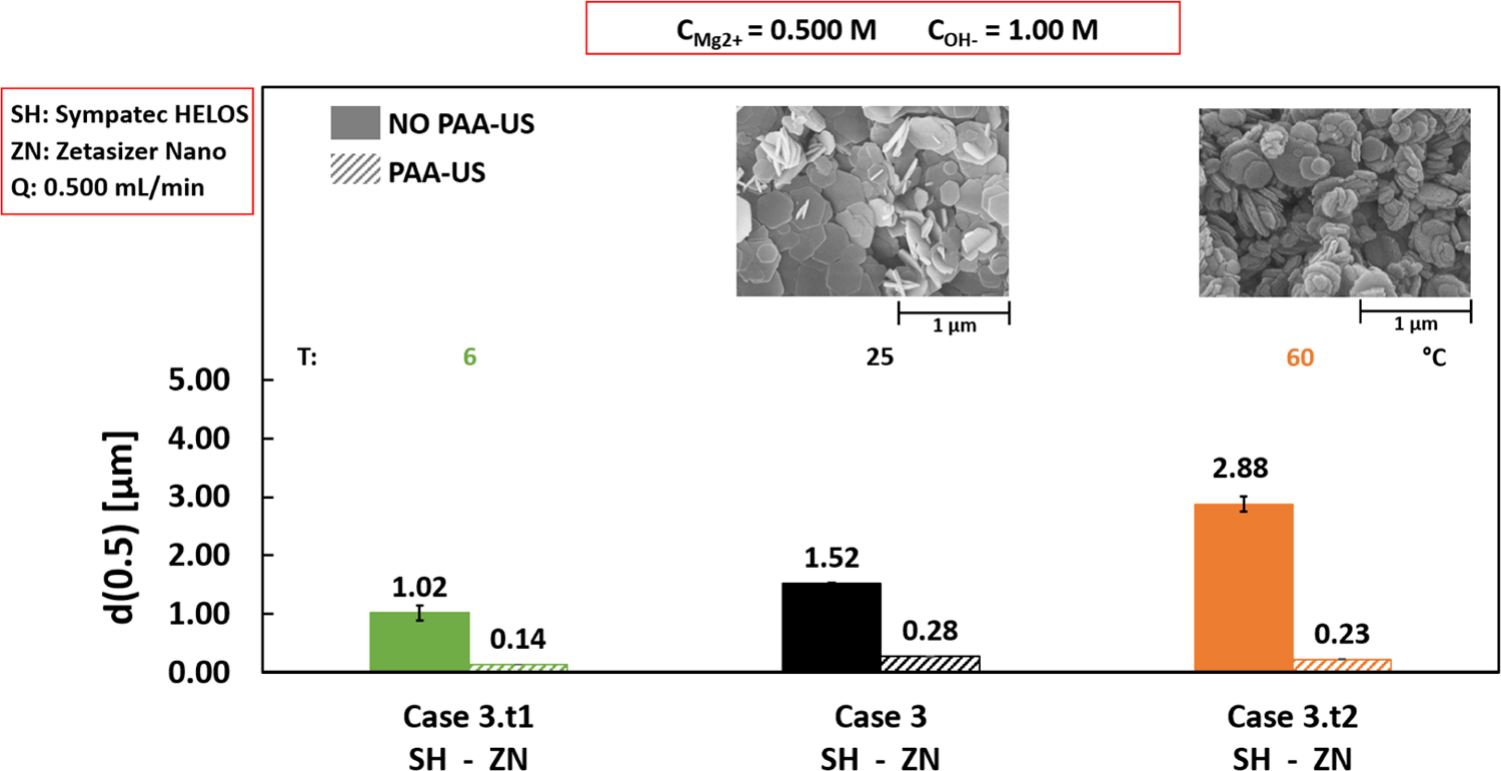
Effect of reaction temperature on median diameter *d*(0.5) values calculated before (solid bars) and after (diagonal stripes bars) PAA-US. 6, 25 and 60 °C were the reaction temperatures set for Cases 3.t1, 3, and 3.t2, respectively. MgCl_2_ and NaOH concentrations = 0.500 and 1 M, flow rates = 0.500 mL/min, stirring speed = 400 rpm. The bottom row indicates the employed particle size analyzer: Sympatec HELOS (SH) or Zetasizer Nano (ZN). The upper row reports SEM images.

Figure 6 reports the median diameters for Cases 3.t1, 3, and 3.t2 conducted at reaction temperatures of 6, 25, and 60 °C, respectively. In all tests, 0.500 M MgCl_2_ and 1.0 M NaOH solutions were used and pumped at 0.500 mL/min.

Before PAA-US (solid bars), agglomerates median diameters increase slightly with the increased reaction temperature. While a not clear trend is noticed after PAA-US (diagonal stripes bars), similar aggregates of ∼0.14, ∼0.28, and ∼0.23 μm are measured for Cases 3.t1 (6 °C), 3 (25 °C), and 3.t2 (60 °C), respectively. Overall, the temperature does not considerably influence the precipitation process, as also confirmed by SEM images. Similar morphology, in fact, is detected for Cases 3 (25 °C) and 3.t2 (60 °C), see Figure 6.

### 3.3 BET Comparison between Particles

A fundamental parameter to identify the industrial suitability of Mg(OH)_2_ powders is the particle’s specific surface area. To assess this parameter, selected samples were analyzed by the BET technique as discussed in Section 2.2. Table 3 reports the obtained values for grown hexagonal Mg(OH)_2_ platelets, from Cases 3.f1 and 3, in double-feed configuration. A comparison of specific surface areas reported in the literature is also presented.

**Table 3:** BET Analyses on Mg(OH)_2_ Powders Obtained in Different Production Routes

authors	production route	BET value	morphology
this work	MgCl_2_ + NaOH at 25 °C in a double-feed stirred tank reactor (Case 3 and Case 3.f1)	∼37 to 45 m^2^/g	hexagonal platelets
Henrist et al.^11^	MgCl_2_ + NH_4_OH at 25 °C in a controlled double-jet stirred tank reactor	∼21 m^2^/g	hexagonal platelets
Henrist et al.^11^	MgCl_2_ + NH_4_OH at 25 °C in controlled double-jet stirred tank reactor 1-week hydrothermal treatment at 170 °C	∼2 m^2^/g	hexagonal platelets
Wu et al.^25^	MgCl_2_ + NaOH at 25 °C in a single-feed stirred tank reactor after the addition of 1 g/L of CaCl_2_ and 4 h hydrothermal treatment at 160 °C	∼29 m^2^/g	hexagonal platelets
Ren et al.^7^	MgCl_2_ + NaOH at 70 °C in T-mixer	∼90 m^2^/g	nanoflakes/globular
Mullin et al.^24^	MgCl_2_ + NaOH at 25 °C in single-feed stirred tank reactor	∼110 to 140 m^2^/g	nanoflakes/globular

Samples produced in Cases 3 and 3.f1 are characterized by specific surface area values ranging from ∼37 to 45 m^2^/g. These values are still 4 times higher than those required for flame-retardant applications (∼10 m^2^/g); however, they are among the lowest ever reported when NaOH is employed to precipitate Mg(OH)_2_ compounds. Considerably higher surface area values were reported by Ren et al.^7^ (∼90 m^2^/g), using a T-mixer, and Mullin et al.^24^ (∼110 to 140 m^2^/g), employing a stirred tank in single-feed mode. These values can be due to high supersaturation levels, high nucleation rate, and thus, a huge number of nanosized particles. As discussed in Section 1, specific surface area values lower than those of Cases 3.f1 and 3 were obtained only after hydrothermal treatment or using NH_4_OH.^11,25^

## 4 Conclusions

The magnesium hydroxide precipitation process, from synthetic MgCl_2_ and NaOH solutions, was experimentally explored in unseeded stirred tank crystallizers in single- and double-feed configurations. The challenge was to identify the best operating conditions to favor crystal growth in a process dominated by nucleation. The influence of reactant flow rates (feeding times), MgCl_2_ and NaOH concentrations, and reaction temperature was analyzed.

In single-feed modes, at the highest flow rates (1 mL/min), the addition of MgCl_2_ solutions into a NaOH bath led to bigger agglomerates than those observed when adding NaOH solutions into the MgCl_2_ bath. This behavior was attributed to the higher reaction pH environment attained in the NaOH bath that promoted particle agglomeration. At the lowest flow rates (0.5 mL/min) (the highest feeding time), larger and stronger agglomerates and aggregates were identified. For these cases, the pH influence was probably overcome by the greater feeding time, which caused a stronger aggregation between primary particles due to the higher particle collision probability in the reactor. The same behavior was observed in double-feed configuration: at decreasing flow rates, increasingly strong agglomerates and aggregates were detected.

In double-feed configuration, (i) at the highest solutions concentration, stronger agglomerates and aggregates were precipitated, probably due to the increase of the solution ionic strength and (ii) no impact of the reaction temperature on agglomerate and aggregate size was observed.

Nanoflakes primary particles with a primary particle size of 50–70 nm were detected in all single-feed experiments, regardless of the reactant feeding configuration or reactant concentrations.

Conversely, well-defined Mg(OH)_2_ hexagonal platelets (especially at low feed flow rates) with a primary particle size of 300–350 nm and characterized by specific surface area values of ∼40 m^2^/g were successfully synthesized in double-feed tests using NaOH solutions. This was achieved thanks to the very accurate control of supersaturation in the system. Specific surface areas reported in the double-feed configurations are still 4 times higher than those required for flame retard applications. Nevertheless, the present work paves the road for a promising precipitation route to produce Mg(OH)_2_ hexagonal platelets using NaOH solutions.

## Figures and Tables

**Figure 7:**
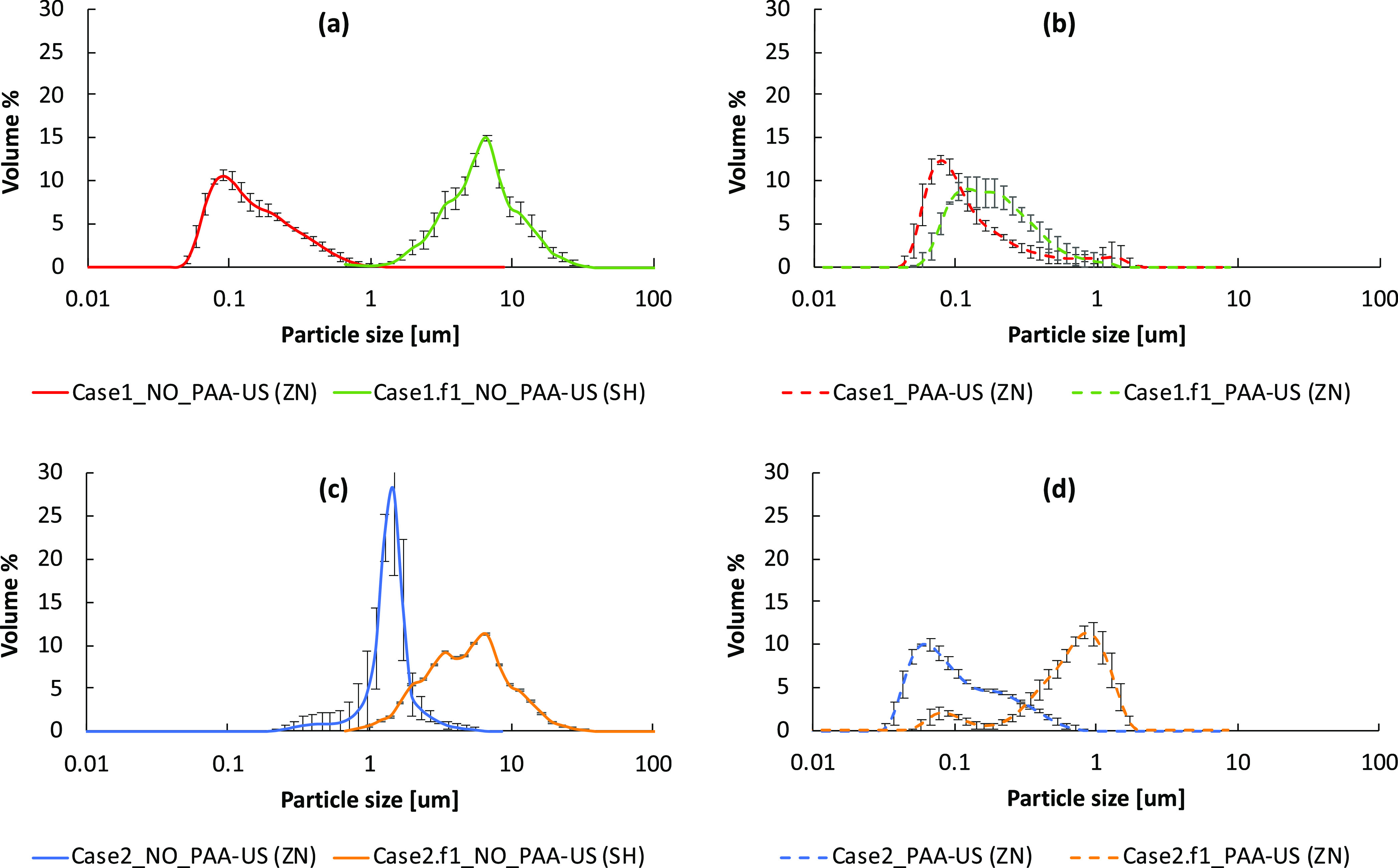
PSDs collected for single-feed cases. Configuration 1 using 0.036 M MgCl_2_ and 0.500 M NaOH (a) before and (b) after PAA-US; Configuration 2 at 0.250 M MgCl_2_ and 0.072 M NaOH (c) before and (d) after PAA-US. In brackets, the employed particle size analyzer: Sympatec HELOS (SH) or Zetasizer Nano (ZN). Feed flow rate = 1.00 mL/min (Cases 1 and 2) and 0.500 mL/min (Cases 1.f1 and 2.f1), stirring speed = 400 rpm, *T* = 25 °C.

**Figure 8:**
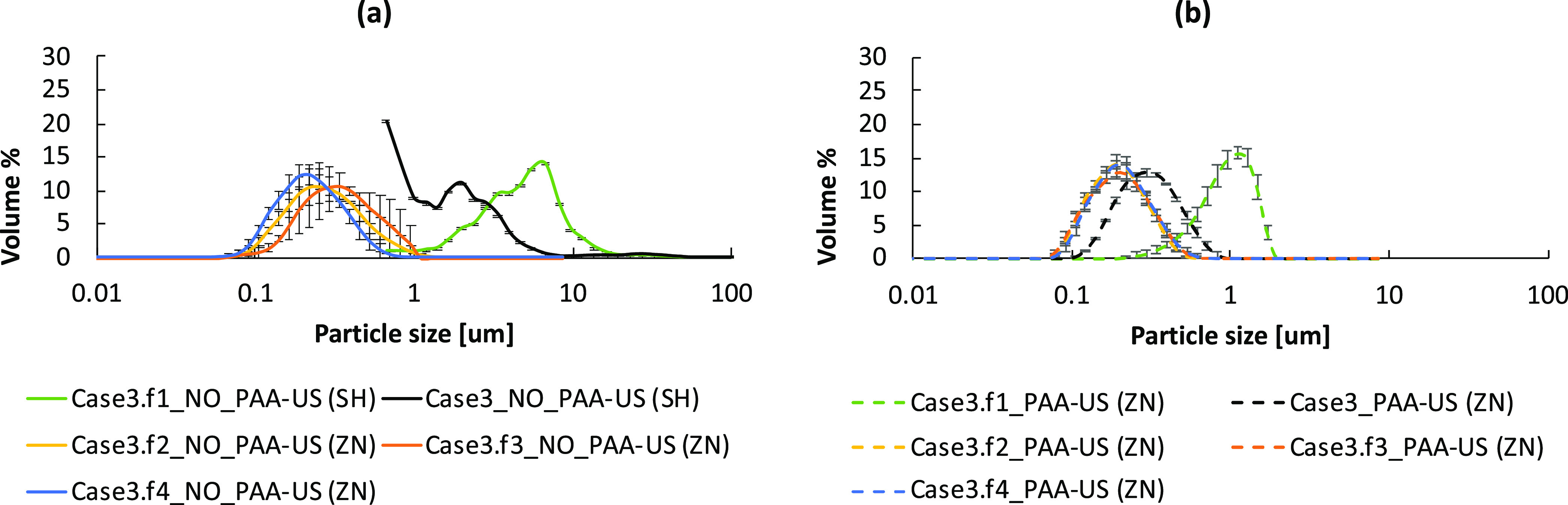
Effect of feeds flow rate on Mg(OH)_2_ particles before (a) and after (b) PAA-US. In brackets, the employed particle size analyzer: Sympatec HELOS (SH) or Zetasizer Nano (ZN). Flow rates of 0.250 mL/min (Case 3.f1), 0.500 mL/min (Case 3), 1.00 mL/min (Case 3.f2), 5 mL/min (Case 3.f3), and 7.50 mL/min (Case 3.f4). MgCl_2_ and NaOH concentrations = 0.500 and 1.00 M, stirring speed = 400 rpm, T = 25 °C.

**Figure 9:**
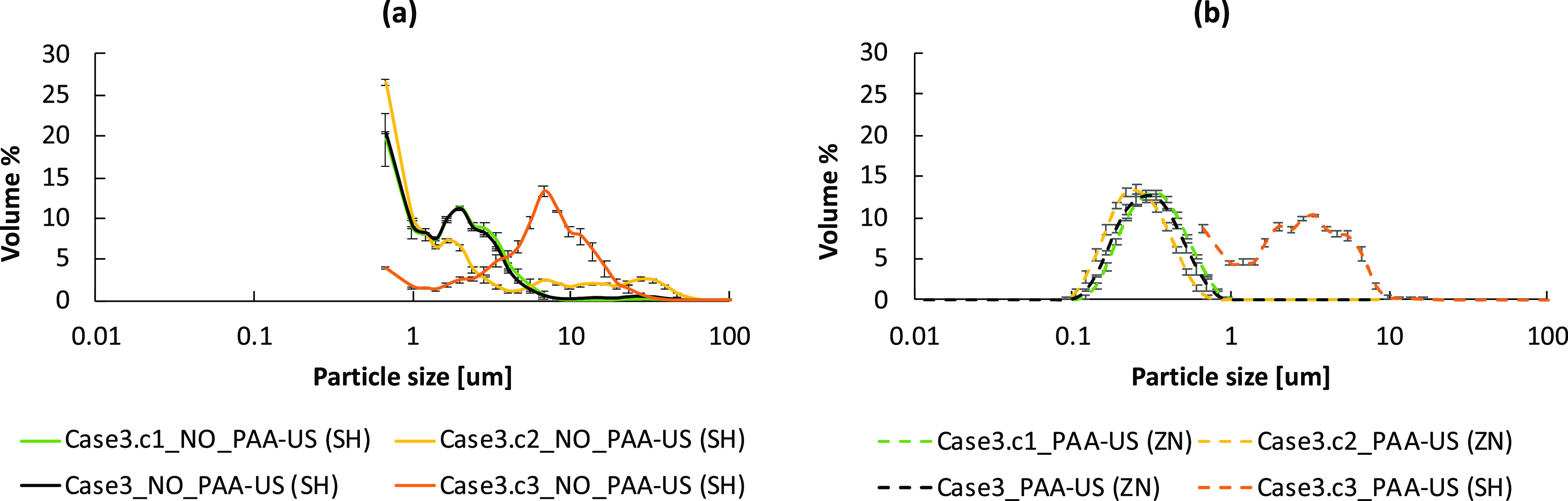
Effect of reactants’ concentrations on Mg(OH)_2_ particles before (a) and after (b) PAA-US. In parentheses, the employed device: Sympatec HELOS (SH) and Zetasizer Nano (ZN). 0.125, 0.250, 0.500, and 1.00 M MgCl_2_ and their NaOH stoichiometric concentration were employed for Cases 3.c1, 3.c2, 3, and 3.c3, respectively. Flow rates = 0.500 mL/min, stirring speed = 400 rpm, T = 25 °C.

**Figure 10:**
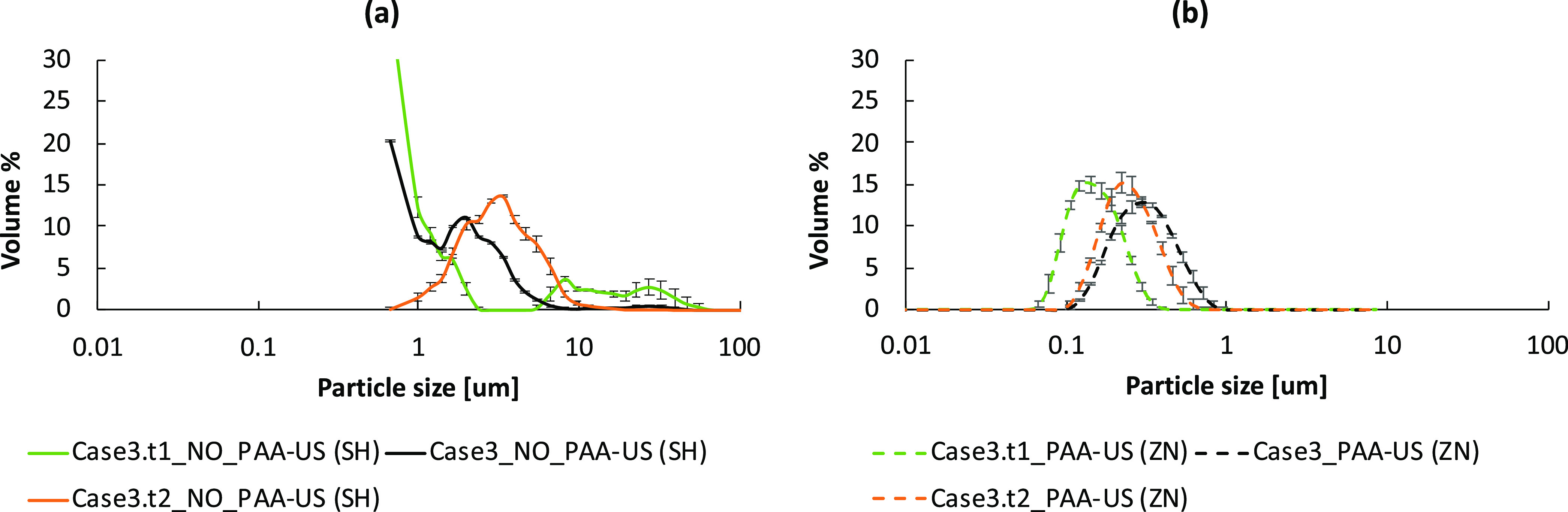
Effect of reaction temperature on Mg(OH)_2_ final particles before (a) and after (b) PAA-US. In parentheses, the employed device: Sympatec HELOS (SH) and Zetasizer Nano (ZN). 6 °C, 25 °C, and 60 °C were the reaction temperatures set for Cases 3.t1, 3, and 3.t2, respectively. MgCl_2_ and NaOH concentrations = 0.500 and 1.00 M, Flow rates = 0.500 mL/min, stirring speed = 400 rpm.

**Figure 11:**
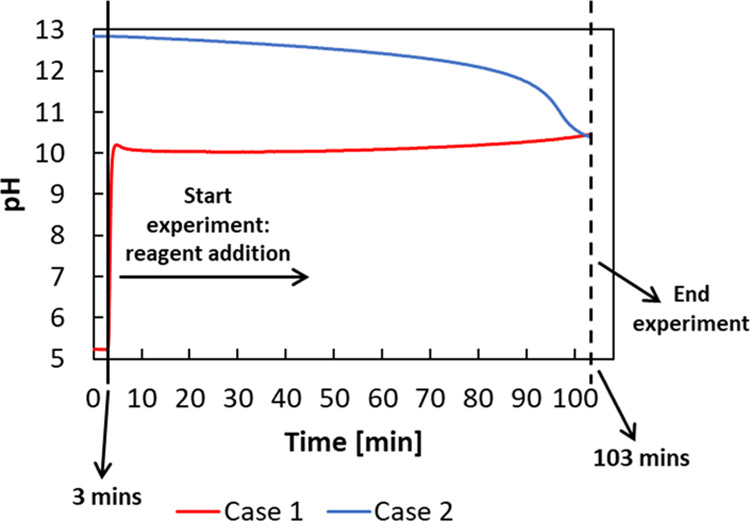
pH profiles along time for Cases 1 (red solid line) and 2 (blue solid line). NaOH solution (Case 1) and MgCl_2_ solution (Case 2) addition began at 3 min and lasted 100 min (final experimental time of 103 min).

**Figure 12:**
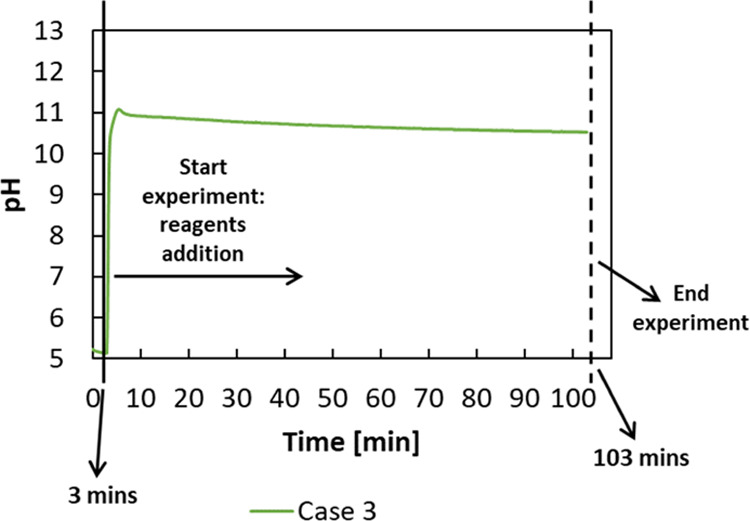
pH profiles along time for Case 3 (green solid line). The addition of MgCl_2_ and NaOH solutions began at 3 min and lasted 100 min (final experimental time of 103 min).

**Figure 13:**
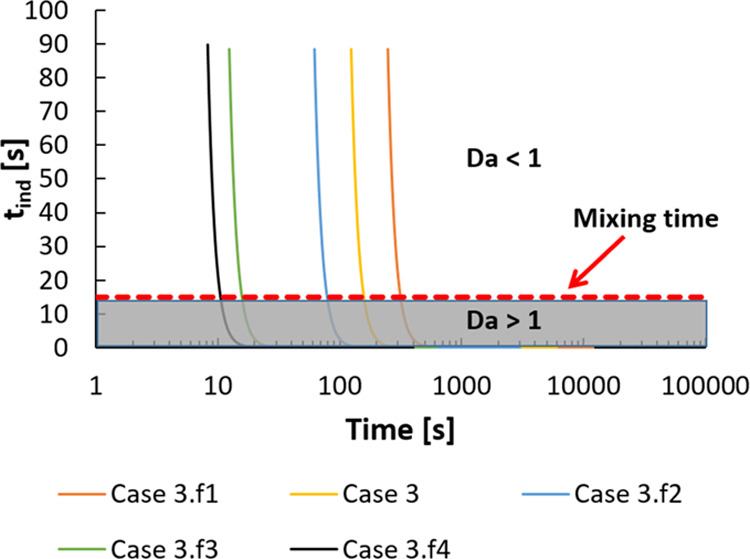
Induction time (*t*_ind_) versus time for Cases 3.f1 (0.250 mL/min), 3 (0.500 mL/min), 3.f2 (1.00 mL/min), 3.f3 (5.00 mL/min), and 3.f4 (7.5 mL/min). MgCl_2_ and NaOH concentrations: 0.500 and 1.00 M; stirring rate = 400 rpm; temperature = 25 °C. A relation between supersaturation and induction time was obtained from experimental data looking at Table 1 of Yuan et al.^18^

**Figure 14:**
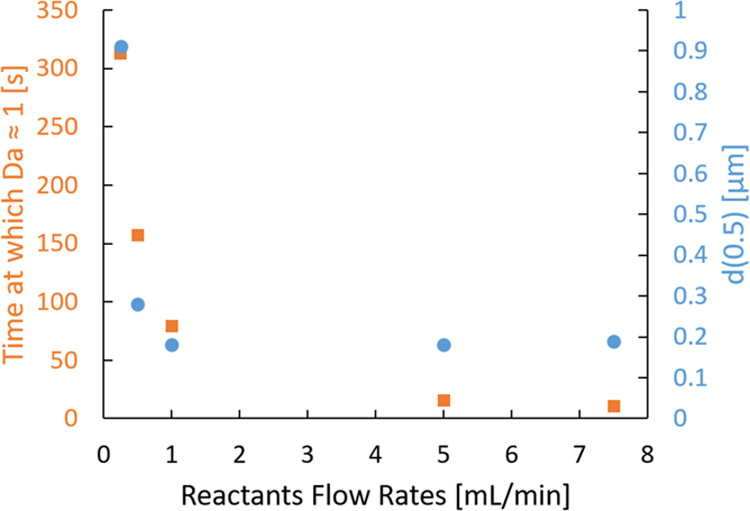
Characteristic diameter, *d*(0.5), values after PAA-US and time at which *Da* ≈ 1 as a function of reactant flow rates for Cases 3.f1 (0.250 mL/min), 3 (0.500 mL/min), 3.f2 (1.00 mL/min), 3.f3 (5.00 mL/min), and 3.f4 (7.5 mL/min). MgCl_2_ and NaOH concentrations: 0.500 and 1.00 M; stirring rate = 400 rpm; temperature = 25 °C. *d*(0.5) shows a plateau already at a reactant flow rate of 1 mL/min. This indicates that the high supersaturation levels achieved in short times promote nucleation phenomena rather than crystal growth, causing a decrease in characteristic diameter *d*(0.5).

## Appendix A

In this appendix, for the sake of completeness, average volume PSDs and characteristic diameters, e.g., *d*(0.1), *d*(0.5), and *d*(0.9), for all discussed cases (Table 1) are reported. In addition, pH profiles measured for Cases 1, 2, and 3 are shown. A preliminary estimation of the influence of mixing time, reactants addition rates, and reaction time on particle size is also provided.

The solid and dashed lines indicate PSDs obtained before and after PAA and ultrasound treatment (PAA-US), respectively. The employed particle size analyzer is reported in parentheses: Sympatec HELOS (SH) or Zetasizer Nano (ZN).

### A.1 PSDs and Characteristic Diameters Collected for Single-Feed Cases (Section 3.2.1)

Figure 7 reports PSD measurements of Cases 1, 1.f1, 2, and 2.f1 in single-feed configuration, before (a, c) and after (b, d) PAA-US treatment.

Tables A1 and AA2 report *d*(0.1), *d*(0.5), and *d*(0.9) calculated from PSDs measurements of Cases 1, 1.f1, 2, and 2.f1 in single-feed configuration, before (Table A1) and after (Table A2) PAA-US.

**Table A1:** *d*(0.1), *d*(0.5), and *d*(0.9) Calculated from PSDs Collected before PAA-US for Cases 1, 1.f1, 2, and 2.f1 in Single-Feed Configuration

	case 1		case 1.f1		case 2		case 2.f1	
	average	STD dev	average	STD dev	average	STD dev	average	STD dev
*d*0.1 [μm]	0.066	0.003	2.620	0.219	0.864	0.266	1.822	0.007
*d*0.5 [μm]	0.120	0.001	5.533	0.540	1.301	0.092	4.428	0.028
*d*0.9 [μm]	0.333	0.036	12.054	1.282	1.792	0.079	10.640	0.171

**Table A2:** *d*(0.1), *d*(0.5), and *d*(0.9) Calculated from PSDs Collected after PAA-US for Cases 1, 1.f1, 2, and 2.f1 in Single-Feed Configuration

	case 1 PAA-US		case 1.f1 PAA-US		case 2 PAA-US		case 2.f1 PAA-US	
	average	STD dev	average	STD dev	average	STD dev	average	STD dev
*d*0.1 [μm]	0.058	0.005	0.082	0.007	0.045	0.002	0.159	0.059
*d*0.5 [μm]	0.097	0.007	0.163	0.008	0.084	0.004	0.660	0.068
*d*0.9 [μm]	0.554	0.333	0.436	0.127	0.268	0.023	1.154	0.078

### A.2 PSDs and Characteristic Diameters Collected for Double-Feed Cases (Section 3.2.2)

#### A.2.1 Influence of Reactant Flow Rate (Section 3.2.2.1)

Figure 8 reports PSD measurements of Cases 3.f1, 3, 3.f2, 3.f3, and 3.f4 in double-feed configuration, before (a) and after (b) PAA-US treatment.

Tables A3 and AA4 report *d*(0.1), *d*(0.5) and *d*(0.9) calculated from PSD measurements of Cases 3.f1, 3, 3.f2, 3.f3, and 3.f4 in double-feed configuration, before (Table A3) and after (Table A4) PAA-US.

**Table A3:** *d*(0.1), *d*(0.5), and *d*(0.9) Calculated from PSDs Collected before PAA-US for Cases 3.f1, 3, 3.f2, 3.f3, and 3.f4 in Double-Feed Configuration^a^

	case 3.f1		case 3		case 3.f2		case 3.f3		case 3.f4	
	average	STD dev	average	STD dev	average	STD dev	average	STD dev	average	STD dev
*d*0.1 [μm]	1.868	0.007			0.131	0.005	0.157	0.008	0.114	0.007
*d*0.5 [μm]	4.397	0.016	1.522	0.007	0.255	0.034	0.318	0.065	0.205	0.014
*d*0.9 [μm]	8.521	0.143	3.496	0.073	0.504	0.129	0.600	0.160	0.375	0.046

a Some *d*(0.1) data are missing because the granulometric analysis resulted in a PSD where the first discrete range of revealed size (bin: 0.5–0.9 μm) is already more than 10% in volume, higher than the *d*(0.1).

**Table A4:** *d*(0.1), *d*(0.5), and *d*(0.9) Calculated from PSDs Collected after PAA-US for Cases 3.f1, 3, 3.f2, 3.f3 and 3.f4 in Double-Feed Configuration

	case 3.f1 PAA-US		case 3 PAA-US		case 3.f2 PAA-US		case 3.f3 PAA-US		case 3.f4 PAA-US	
	average	STD dev	average	STD dev	average	STD dev	average	STD dev	average	STD dev
*d*0.1 [μm]	0.498	0.037	0.163	0.001	0.108	0.004	0.106	0.006	0.114	0.007
*d*0.5 [μm]	0.914	0.039	0.280	0.007	0.179	0.005	0.184	0.006	0.189	0.001
*d*0.9 [μm]	1.357	0.047	0.488	0.025	0.301	0.001	0.324	0.006	0.329	0.030

#### A.2.2 Influence of Reactant Concentrations (Section 3.2.2.2)

Figure 9 reports PSD measurements of Cases 3.c1, 3.c2, 3, and 3.c3 in double-feed configuration, before (a) and after (b) PAA-US treatment.

Tables A5 and AA6 report *d*(0.1), *d*(0.5), and *d*(0.9) calculated from PSD measurements of Cases 3.c1, 3.c2, 3, and 3.c3 in double-feed configuration, before (Table A5) and after (Table A6) PAA-US.

**Table A5:** *d*(0.1), *d*(0.5), and *d*(0.9) Calculated from PSDs Collected before PAA-US for Cases 3.c1, 3.c2, 3, and 3.c3 in Double-Feed Configuration^a^

	case 3.c1		case 3.c2		case 3		case 3.c3	
	average	STD dev	average	STD dev	average	STD dev	average	STD dev
*d*0.1 [μm]							1.606	0.080
*d*0.5 [μm]	1.563	0.152	1.372	0.019	1.522	0.007	5.926	0.445
*d*0.9 [μm]	3.439	0.360	3.474	0.125	3.496	0.073	13.019	1.141

a Some *d*(0.1) data are missing because the granulometric analysis resulted in a PSD where the first discrete range of revealed size (bin: 0.5–0.9 μm) is already more than 10% in volume, higher than the *d*(0.1).

**Table A6:** *d*(0.1), *d*(0.5), and *d*(0.9) Calculated from PSDs Collected before PAA-US for Cases 3.c1, 3.c2, 3, and 3.c3 in Double-Feed Configuration

	case 3.c1 PAA-US		case 3.c2 PAA-US		case 3 PAA-US		case 3.c3 PAA-US	
	average	STD dev	average	STD dev	average	STD dev	average	STD dev
*d*0.1 [μm]	0.178	0.005	0.143	0.006	0.163	0.001	0.763	0.044
*d*0.5 [μm]	0.304	0.006	0.239	0.006	0.280	0.007	2.532	0.130
*d*0.9 [μm]	0.525	0.005	0.409	0.013	0.488	0.025	5.302	0.271

#### A.2.3 Influence of Reactant Concentrations (Section 3.2.2.3)

Figure 10 reports PSD measurements of Cases 3.t1, 3, and 3.t2 in double-feed configuration, before (a) and after (b) PAA-US treatment.

Tables A7 and AA8 report *d*(0.1), *d*(0.5), and *d*(0.9) calculated from PSDs measurements of Cases 3.t1, 3, and 3.t2 in double-feed configuration, before (Table A7) and after (Table A8) PAA-US.

**Table A7:** *d*(0.1), *d*(0.5), and *d*(0.9) Calculated from PSDs Collected before PAA-US for Cases 3.t1, 3, and 3.t2 in Double-Feed Configuration^a^

	case 3.t1		case 3		case 3.t2	
	average	STD dev	average	STD dev	average	STD dev
*d*0.1 [μm]					1.468	0.063
*d*0.5 [μm]	1.018	0.134	1.522	0.007	2.876	0.130
*d*0.9 [μm]	4.902	0.099	3.496	0.073	5.390	0.160

a Some *d*(0.1) data are missing because the granulometric analysis resulted in a PSD where the first discrete range of revealed size (bin: 0.5–0.9 μm) is already more than 10% in volume, thus preventing from having the *d*(0.1).

**Table A8:** *d*(0.1), *d*(0.5), and *d*(0.9) Calculated from PSDs Collected before PAA-US for Cases 3.t1, 3, and 3.t2 in Double-Feed Configuration

	case 3.t1 PAA-US		case 3 PAA-US		case 3.t2 PAA-US	
	average	STD dev	average	STD dev	average	STD dev
*d*0.1 [μm]	0.089	0.004	0.163	0.001	0.143	0.003
*d*0.5 [μm]	0.137	0.004	0.280	0.007	0.225	0.007
*d*0.9 [μm]	0.221	0.006	0.488	0.025	0.369	0.029

### A.3 pH Profiles of Cases 1, 2, and 3

pH profiles were collected in Cases 1, 2, and 3. Figure 11 shows the pH profiles of Cases 1 and 2 in single-feed configuration.

As can be observed, at the beginning of the feed addition, the initial measured pH values were about 10 and 13 for Case 1 (red solid line) and Case 2 (blue solid line), respectively, due to the initial solution contained within the reactor, i.e., MgCl_2_ for Case 1 and NaOH for Case 2.

pH slowly increased from 10 to 10.5 in Case 1, while it slowly decreased from 13 to 12 and then rapidly, at the end of the test, to 10.5 in Case 2. In both cases, the final measured pH value of 10.5, close to the equilibrium one, indicated the complete conversion of the reagents.

In addition, the pH profile of Case 3 in double-feed configuration is shown in Figure 12.

As can be seen, pH profiles rapidly increased from 5 to 10 after reagent addition, slowly reaching 11, before decreasing to the equilibrium pH value of 10.5. It is worth noting that a similar pH profile was found in Case 1, see Figure 11, but, as widely discussed in Section 3.1, the reagents drop dilution, before the reaction, changes depending on the feeding configurations. Therefore, the local pH measurement in a double-feed configuration is closer to the reaction environment due to higher reagents homogenization before reacting.

### A.4 Preliminary Estimation of the Influence of Mixing Time, Reactants Addition Rates, and Reaction Time on Particles Size

A preliminary analysis on the influence of mixing, reagents addition, and reaction times on particle sizes was carried out considering the results obtained for Cases 3.f1, 3, 3.f2, 3.f3, and 3.f4 (reagents flow rates of 0.250, 0.500, 1.00, 5.00 and 7.50 mL/min, respectively). The concentrations of MgCl_2_ and NaOH solutions were always 0.500 and 1.00 M.

In precipitation processes, the Damköhler number is the timescale ratio between the mixing and solid formation (reaction time)

2

where *t*_m_ is the mixing time and *t*_r_ is the chemical reaction time. Both mixing and reaction times can be better addressed by process modeling of a system. In the present work, a preliminary estimation is conducted. The reaction time was assumed to be equal to the induction one (*t*_ind_), which is the time that elapses between the onset of supersaturation and the formation of a solid phase. This assumption is likely an underestimation, as the experimental induction time is typically longer than the reaction one (solids must reach a detectable size to be observed^40^). However, the induction time was adopted since experimental data with respect to supersaturation levels are available in the literature.^18,41^ The mixing time, as discussed in Section 2.1, was estimated to be equal to 15 s.

Nevertheless, the supersaturation value inside the tank was determined by assuming a perfect mixing condition at each instant of experimental tests; consequently, the induction time is evaluated for each supersaturation value.

Figure 13 shows the induction time (*t*_ind_) trends as a function of the time estimated for Cases 3.f1, 3, 3.f2, 3.f3, and 3.f4.

In Figure 13, two regions are delineated by a red horizontal dashed line (*Da* ≈ 1). Above the dashed line, the induction time is higher than the mixing time (*Da* < 1) and thus the precipitation rate is the rate-determining step; conversely, below, the precipitation rate becomes faster than the mixing (*Da* > 1). In this latter region, mixing is the rate-determining step that is not enough adequate to ensure a good reactants homogenization before the reaction (possible local supersaturation hotspot).

At increasing flow rates, the time at which *Da* ≈ 1 decreases due to the faster reactants addition rate and thus the higher supersaturation level, as can be clearly observed in Figure 14.

Figure 14 shows also the characteristic diameter, *d*(0.5), values with PAA-US as a function of reactant flow rates.

## Supporting Information Available

Enlarged images of SEM analyses; comparison between particle size distributions for Cases 3 and 3.c1 obtained by adopting the SYMPATEC HELOS and the Zetasizer Nano devices; SEM micrographs at different locations of the same sample for Cases 1.f1, 1, 3, and 3.f4 (SEM statistical relevance); and repeatability and reproducibility analyses (PDF)

## Abbreviations

PAA: poly(acryclic acid, sodium salt)
PSD: particle size distribution
US: ultrasound treatment
SLS: static light scattering
DLS: dynamic light scattering
SEM: scanning electron microscopy technique
BET: Brunauer–Emmett–Teller
SH: SYMPATEC HELOS granulometer
ZN: Zetasizer Nano ZS
