# Supplementary material for: The Role of Operating Conditions in the Precipitation of Magnesium Hydroxide Hexagonal Platelets Using NaOH Solutions
Source: Cryst Growth Des. 2023 Aug 8;23(9):6491–505. doi: 10.1021/acs.cgd.3c00462 (PMC10485817; doi:10.1021/acs.cgd.3c00462)
Supplement: Supplementary file 1 — cg3c00462_si_001.pdf [file cg3c00462_si_001.pdf]

Supporting Information

The role of operating conditions in the precipitation of magnesium hydroxide hexagonal platelets using NaOH solutions

Salvatore Romano†, Silvio Trespi‡, Ramona Achermann‡, Giuseppe Battaglia*,†, Antonello Raponi§, Daniele Marchisio§, Marco Mazzotti‡, Giorgio Micale†, Andrea Cipollina†

† Università degli studi di Palermo, Dipartimento di Ingegneria, Viale delle Scienze, 90128 Palermo, Italy

‡ Institute of Energy and Process Engineering, ETH Zurich, 8092 Zurich, Switzerland

§ Department of Applied Science and Technology, Institute of Chemical Engineering, Politecnico di Torino, Torino 10129, Italy

**Email*: giuseppe.battaglia03@unipa.it

S.1 ENLARGED SEM PICTURES

Figure S1 reports enlarged SEM images of Cases 1 (a) and 1.f1 (b) in single-feed configuration.

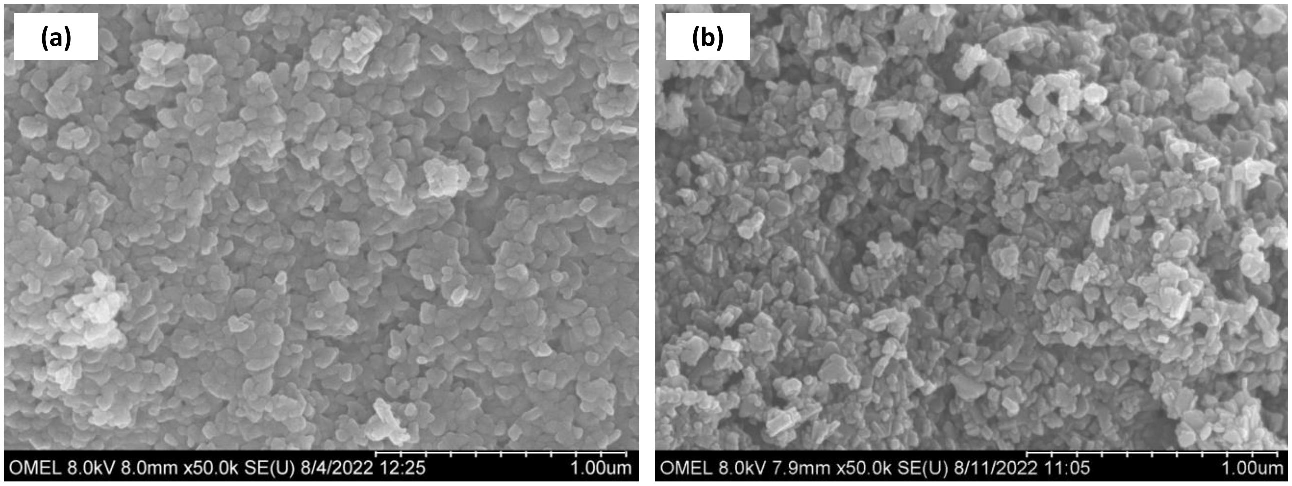

Figure S1. SEM pictures collected for Case 1 (a) and Case 1.f1 (b). $MgCl_2$ and NaOH concentrations = 0.036 M and 0.500 M, feed flow rate = 1.00 mL/min (Case 1) and 0.500 mL/min (Case 1.f1), stirring speed = 400 rpm, T = 25 °C.

Figure S2 reports enlarged SEM images of Cases 3.f1 (a), 3 (b), 3.f2 (c), 3.f3 (d) and 3.f4 (e) in double-feed configuration.

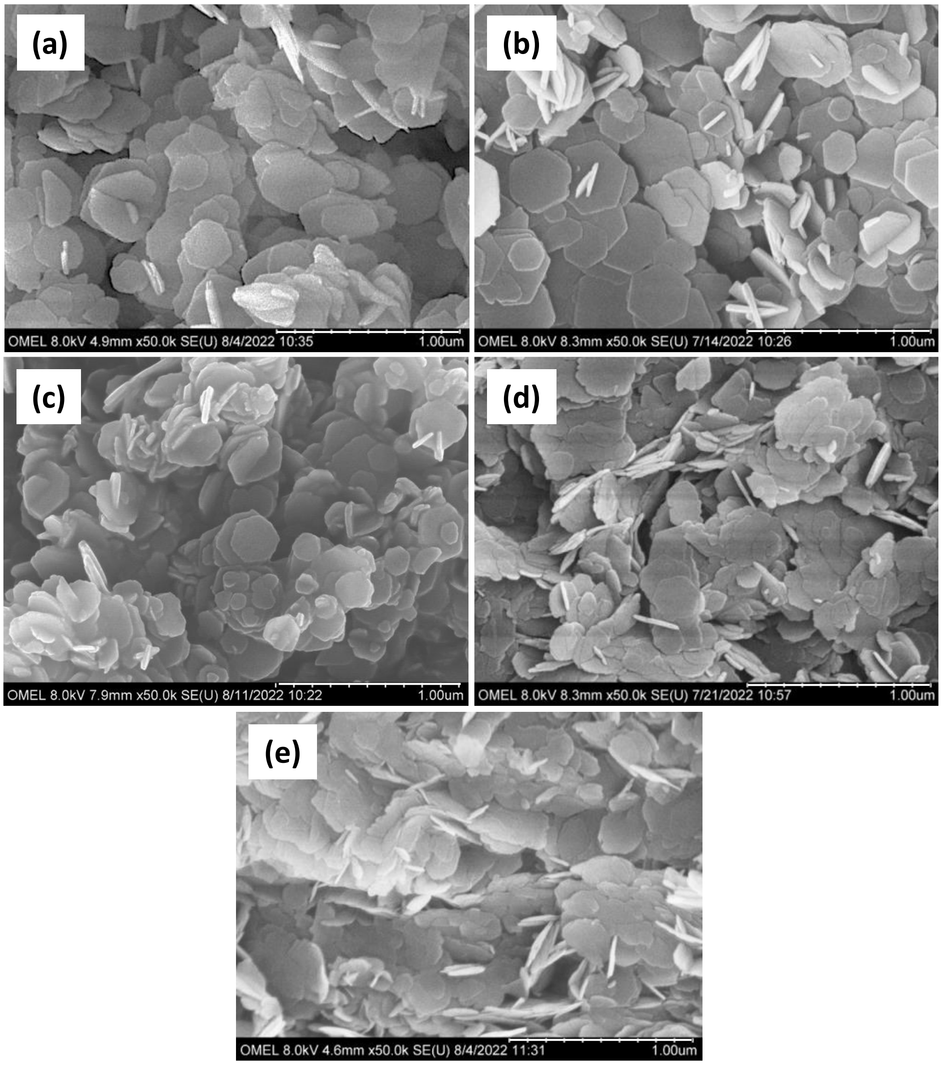

Figure S2. SEM pictures collected for cases at different feeds flow rates: Case 3.f1 (a), Case3 (b), Case 3.f2 (c), Case 3.f3 (d) and Case 3.f4 (e). Flow rates of 0.250 mL/min (Case 3.f1), 0.500 mL/min (Case 3), 1.00 mL/min (Case 3.f2), 5.00 mL/min (Case 3.f3) and 7.50 mL/min (Case 3.f4). $MgCl_2$ and NaOH concentrations = 0.500 and 1.00 M, stirring speed = 400 rpm, T = 25 °C.

Figure S3 reports enlarged SEM images of Cases 3 (a) and 3.c3 (b) in double-feed configuration.

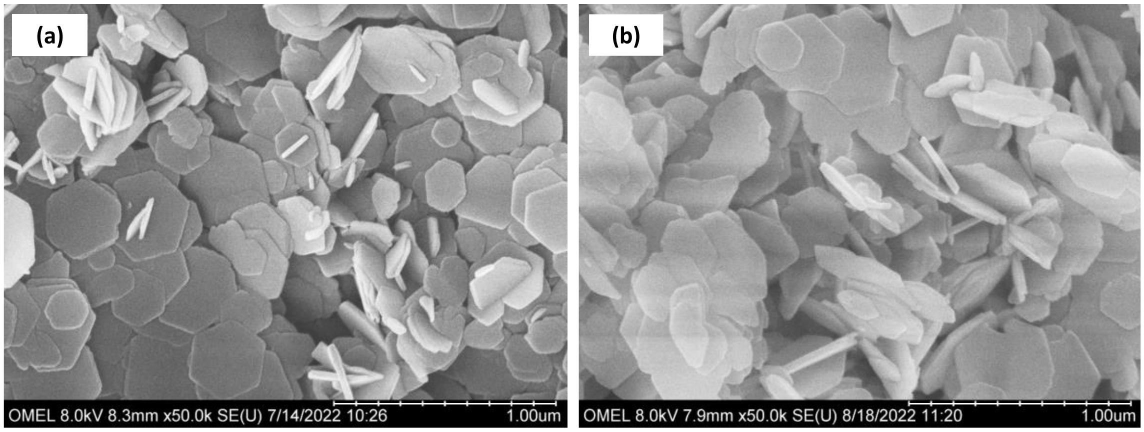

Figure S3. SEM pictures collected for Case 3 (a) and Case 3.c3 (b). $MgCl_2$ and NaOH concentrations = 0.500 M and 1.00 M (Case 3); 1.00 M and 2.00 M (Case 3.c3), flow rates = 0.500 mL/min, stirring speed = 400 rpm, T = 25 °C.

Figure S4 reports enlarged SEM images of Cases 3 (a) and 3.t2 (b) in double-feed configuration.

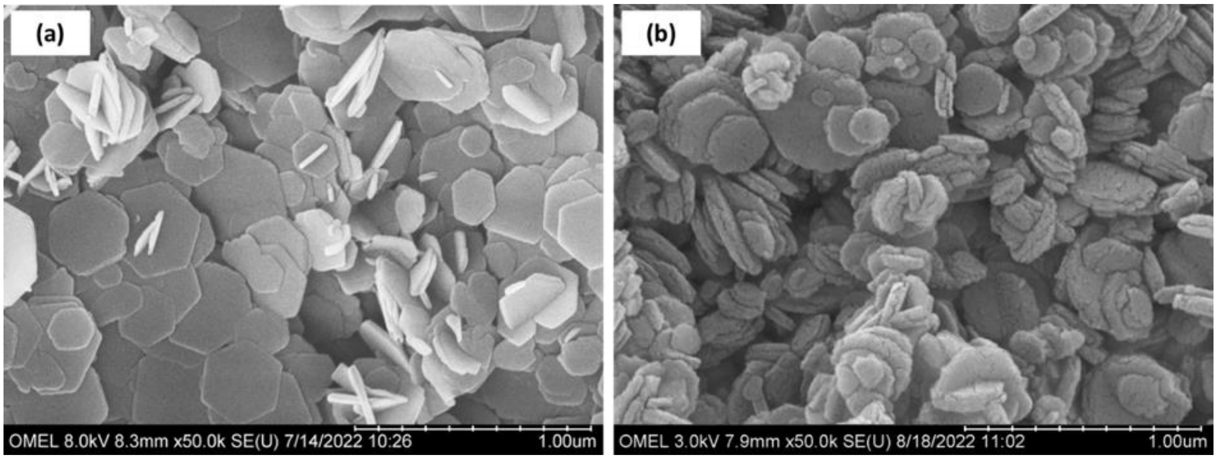

Figure S4. SEM pictures collected for Case 3 (a) and Case 3.t2 (b). T = 25 °C (Case 3); T = 60 °C (Case 3.t2); $MgCl_2$ and NaOH concentrations = 0.500 M and 1.00 M; flow rates = 0.500 mL/min, stirring speed = 400 rpm.

S.2 PARTICLE SIZE DISTRIBUTIONS ANALYSIS BY SYMPATEC HELOS GRANULOMETER AND ZETASIZER NANO

The SYMPATEC HELOS granulometer and the Zetasizer Nano ZS are based on static light scattering and dynamic light scattering techniques, respectively. Different results can be obtained analyzing the same sample by the two equipment. To investigate a possible offset between the measurements, Figure S5 reports Particle Size Distributions (PSDs) of $Mg(OH)_2$ particles collected for Case 3 and Case 3.c1. PSDs were obtained by using the SYMPATEC HELOS granulometer (green solid lines) and the Zetasizer Nano ZS (dashed orange lines) without the addition of PAA and the ultrasound treatment. The particle size of 1 μm can be considered as a common limit between the two equipment.

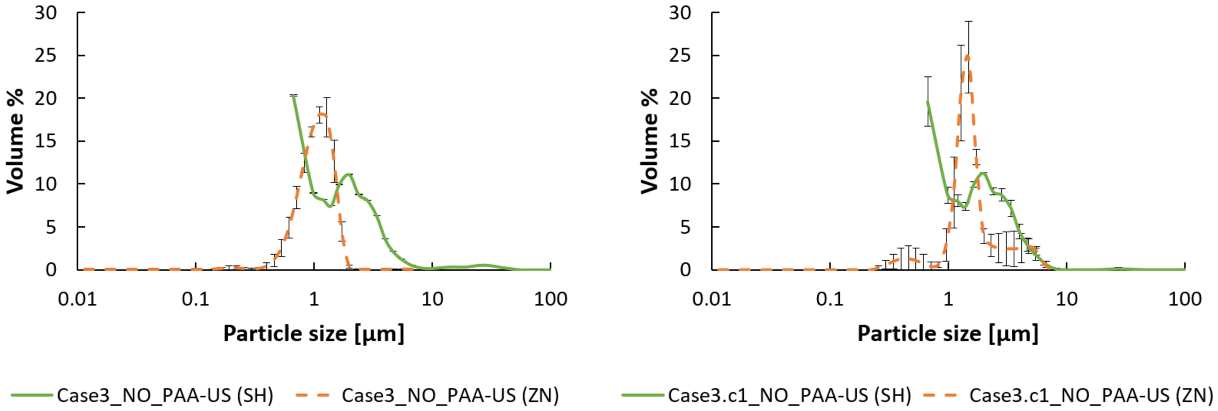

Figure S5. PSDs measured employing Sympatec HELOS (SH), green solid line, and Zetasizer Nano (ZN), dashed orange line, for Cases 3 (left) and 3.c1 (right) before PAA-US. $MgCl_2$ concentrations = 0.500 M (Case 3) and 0.125 M (Case 3.c1), NaOH in stoichiometric amount; reagents flow rates = 0.500 mL/min, stirring speed = 400 rpm, T = 25 °C.

PSDs obtained by the two equipment are almost overlapping. However, the SH well characterizes particles of bigger sizes, while the ZN, particles sizes lower than 1 μm. These results

are expected due to the different particle size range of the two adopted techniques. Table S1 reports the d(0,1), d(0,5) and d(0,9) for the above discussed cases.

	**Case3_NO_PAA-US (SH)**		**Case3_NO_PAA-US (ZN)**	
	Average	STD dev	Average	STD dev
**d(0.1) [µm]**	---	---	0.692	0.057
**d(0.5) [µm]**	1.522	0.007	0.983	0.057
**d(0.9) [µm]**	3.496	0.073	1.394	0.038
	**Case3.c1_NO_PAA-US (SH)**		**Case3.c1_NO_PAA-US (ZN)**	
	Average	STD dev	Average	STD dev
**d(0.1) [µm]**	---	---	0.826	0.275
**d(0.5) [µm]**	1.563	0.152	1.371	0.057
**d(0.9) [µm]**	3.439	0.360	2.912	0.888

Table S1. Characteristic diameters, i.e. d(0.1), d(0.5) and d(0.9), obtained from PSDs measured employing Sympatec HELOS (SH), and Zetasizer Nano (ZN), for Cases 3 and 3.c1 before PAA-US.

d(0.5) values show an offset between the two techniques. However, due to the presence of bigger particles in the samples, SH measurements provide a better estimation of the sample PSDs.

S.3 SEM MICROGRAPHS

Two SEM micrographs at different locations of the same sample were provided for Cases 1.f1, 1, 3 and 3.f4 to confirm the observations discussed in the main manuscript. Figures S6, S7, S8 and S9, show the SEM micrographs for Cases 1.f1, 1, 3 and 3.f4, respectively.

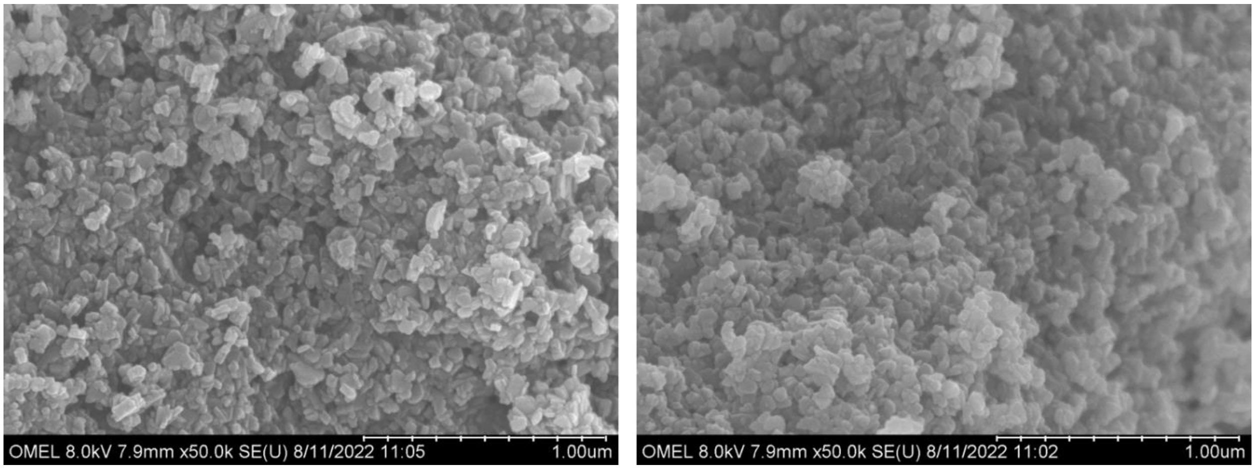

Figure S6. SEM pictures collected for Case 1.f1 in two different sites of the same sample. $MgCl_2$ and NaOH concentrations = 0.036 M and 0.500 M, feed flow rate = 0.500 mL/min, stirring speed = 400 rpm, T = 25 °C.

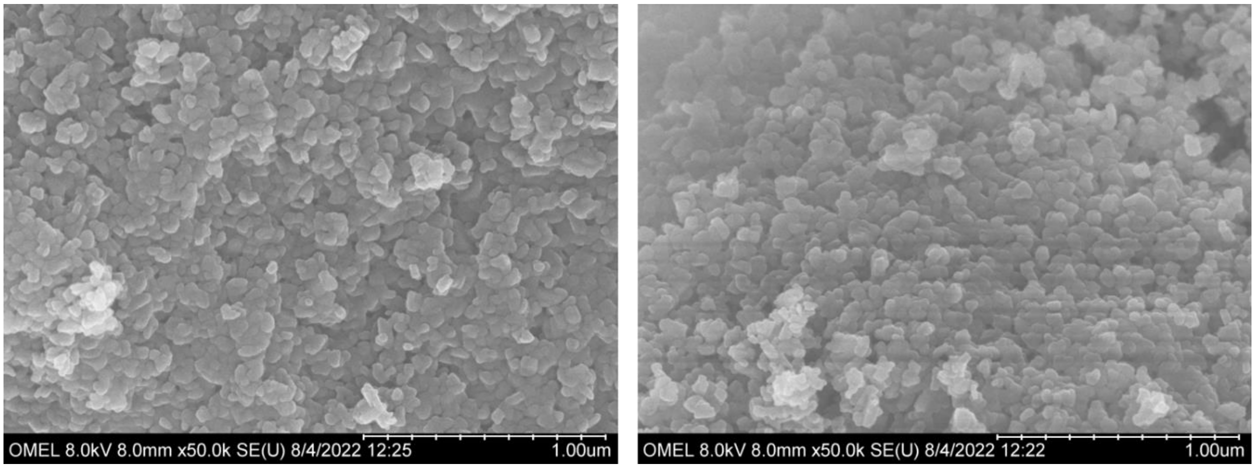

Figure S7. SEM pictures collected for Case 1 in two different sites of the same sample. $MgCl_2$ and NaOH concentrations = 0.036 M and 0.500 M, feed flow rate = 1.00 mL/min, stirring speed = 400 rpm, T = 25 °C.

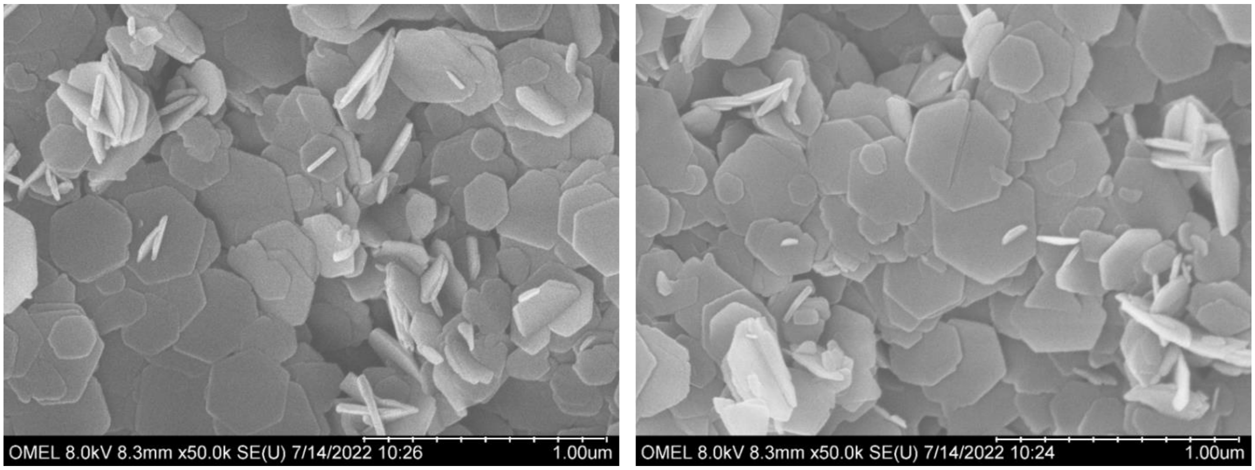

Figure S8. SEM pictures collected for Case 3 in two different sites of the same sample. Feeds flow rates of 0.500 mL/min, $MgCl_2$ and NaOH concentrations = 0.500 M and 1.00 M, stirring speed = 400 rpm, T = 25 °C.

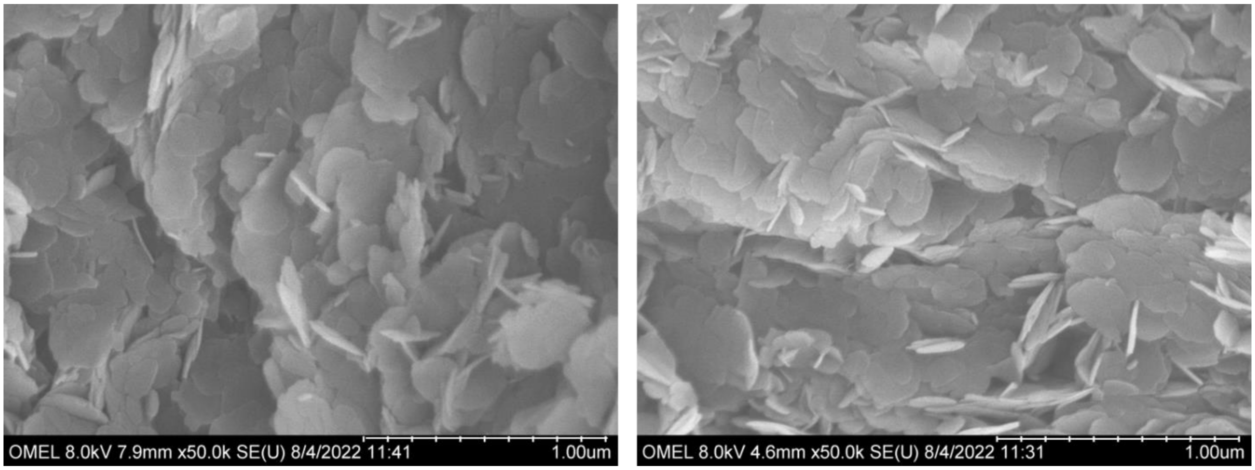

Figure S9. SEM pictures collected for Case 3.f4 in two different sites of the sample. Feeds flow rates of 7.50 mL/min, $MgCl_2$ and NaOH concentrations = 0.500 M and 1.00 M, stirring speed = 400 rpm, T = 25 °C.

S.4 REPEATABILITY AND REPRODUCIBILITY

The repeatability and reproducibility of the experimental results were addressed by considering Case 3. Concerning the repeatability, Figure S10 shows PSDs collected before PAA-US, 5 measurements using Sympatec HELOS granulometer, and after PAA-US, 3 measurements using Zetasizer. Measurements were carried out two times on fresh $Mg(OH)_2$ suspensions (produced 3 weeks after).

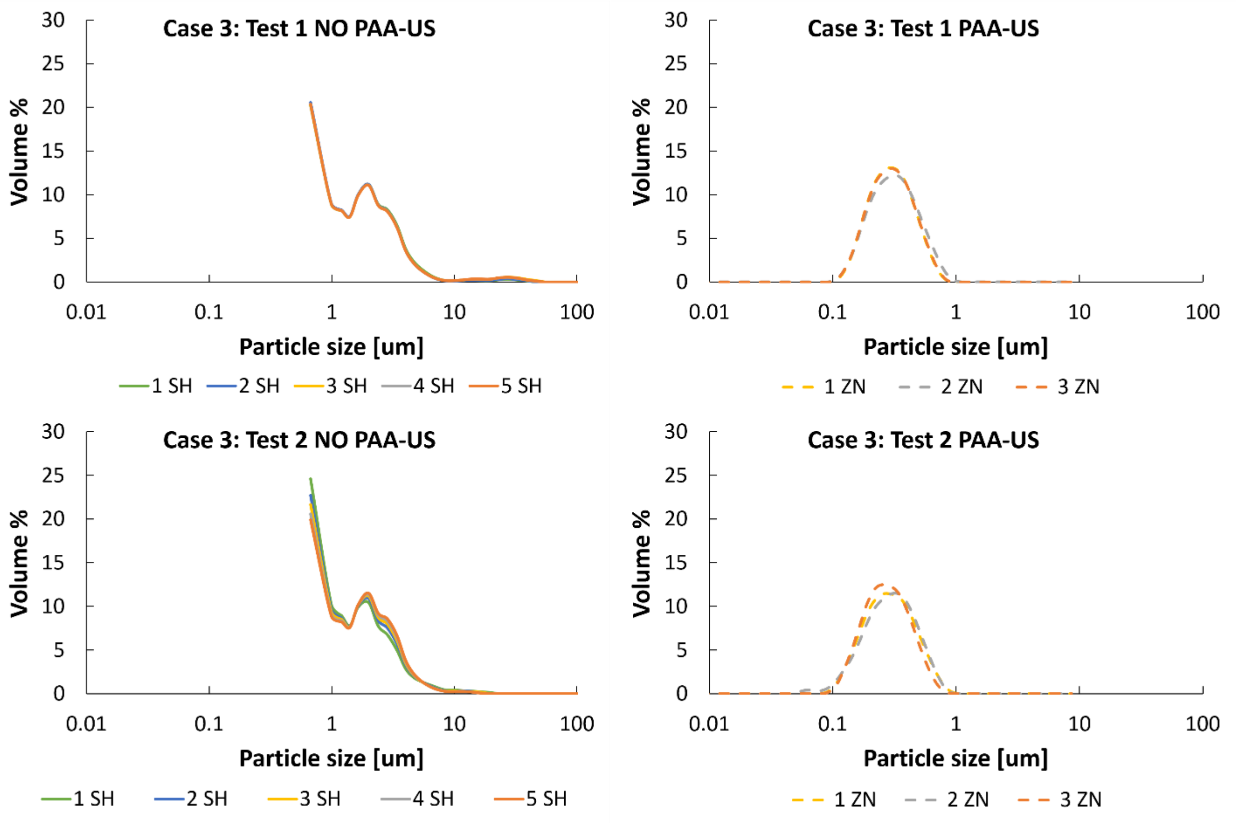

Figure S10. Repeatability analysis. On the left, 5 measurements (SH) before PAA-US; on the right, 3 measurements (ZN) after PAA-US for fresh $Mg(OH)_2$ suspensions (Test 1 and Test 2 performed 3 week after) produced under the operating conditions of Cases 3. In the legend, SH

and ZN indicate the employed particle size analyzer: Sympatec HELOS and Zetasizer Nano, respectively.

PSDs very well overlap each other, indicating high repeatability of the experimental data. Average d(0.1), d(0.5) and d(0.9) among measurements are reported in Table S2.

	**Test 1 NO PAA-US (SH)**			**Test 1 PAA-US (ZN)**		
	Average	STD dev	COV %	Average	STD dev	COV %
d(0.1) [μm]	---	---	---	0.163	0.001	0.5
d(0.5) [μm]	1.522	0.007	0.5	0.280	0.007	2.5
d(0.9) [μm]	3.469	0.073	2.1	0.488	0.025	5.1
	**Test 2 NO PAA-US (SH)**			**Test 2 PAA-US (ZN)**		
	Average	STD dev	COV %	Average	STD dev	COV %
d(0.1) [μm]	---	---	---	0.145	0.003	1.9
d(0.5) [μm]	1.458	0.065	4.5	0.264	0.011	4.1
d(0.9) [μm]	3.284	0.031	0.9	0.477	0.028	5.9

Table S2. Characteristic diameters, i.e. d(0.1), d(0.5) and d(0.9), obtained from PSDs measured employing Sympatec HELOS (SH) and Zetasizer Nano (ZN) for Tests 1 and 2 of Case 3 before and after PAA-US. The COV was calculated as the ratio between the average diameter and its standard deviation among each measurement.

The coefficient of variation (COV) of the characteristic diameters is always below 6 % showing an excellent repeatability of the experimental data.

Figure S10 and data in Table S2 also clearly show excellent reproducibility of the experimental data. Characteristic values differ by less than 5 % from each other. Only d(1,0) shows a relative

difference of about 10 %. Figure S11 also shows an excellent overlapping between average PSDs for Tests 1 and 2 before and after PAA-US.

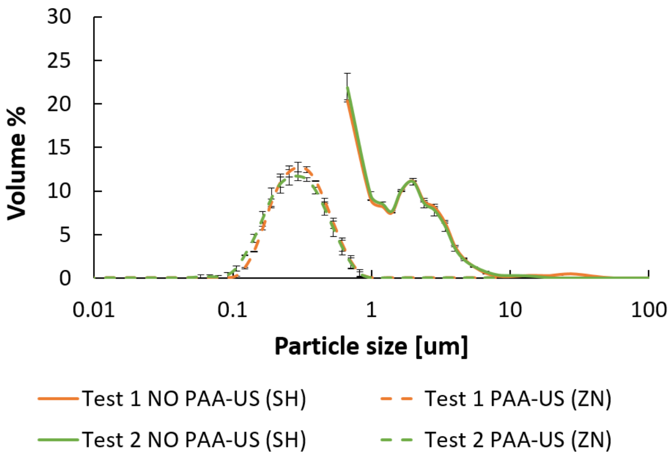

Figure S11. Reproducibility analysis on Case 3 conducted at two different times: Test 1 and Test 2 (performed after about 3 weeks). Average PSD was shown for Tests 1 (orange lines) and 2 (green lines) before (solid lines) and after (dashed lines) PAA-US. In the legend, SH and ZN indicate the employed particle size analyzer: Sympatec HELOS and Zetasizer Nano, respectively.

Morphology data reproducibility was also assessed. Figure S12 shows the SEM images of Case 3-Test 1 and Case 3-Test 2 (performed 3 weeks later).

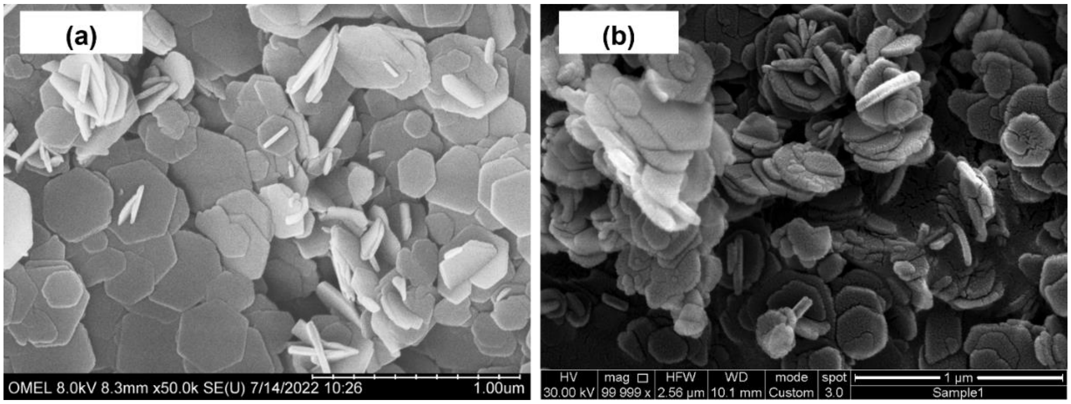

Figure S12. Reproducibility analysis on Case 3 replicates: SEM images of (a) Case 3-Test 1 and (b) Case 3-Test2 (performed 3 weeks later). SEM images were taken by using the Hitachi S-4800 (at ETH laboratories, figure a), and the FEI Quanta 200 FEG (at UNIPA laboratories, figure b).

SEM images confirmed the high reproducibility of the experimental data.
